# Chromosome I Controls Chromosome II Replication in *Vibrio cholerae*

**DOI:** 10.1371/journal.pgen.1004184

**Published:** 2014-02-27

**Authors:** Jong Hwan Baek, Dhruba K. Chattoraj

**Affiliations:** Laboratory of Biochemistry and Molecular Biology, Center for Cancer Research, National Cancer Institute, National Institutes of Health, Bethesda, Maryland, United States of America; Institute of Molecular and Cell Biology (IMCB), A*STAR, Singapore

## Abstract

Control of chromosome replication involves a common set of regulators in eukaryotes, whereas bacteria with divided genomes use chromosome-specific regulators. How bacterial chromosomes might communicate for replication is not known. In *Vibrio cholerae*, which has two chromosomes (chrI and chrII), replication initiation is controlled by DnaA in chrI and by RctB in chrII. DnaA has binding sites at the chrI origin of replication as well as outside the origin. RctB likewise binds at the chrII origin and, as shown here, to external sites. The binding to the external sites in chrII inhibits chrII replication. A new kind of site was found in chrI that enhances chrII replication. Consistent with its enhancing activity, the chrI site increased RctB binding to those chrII origin sites that stimulate replication and decreased binding to other sites that inhibit replication. The differential effect on binding suggests that the new site remodels RctB. The chaperone-like activity of the site is supported by the finding that it could relieve the dependence of chrII replication on chaperone proteins DnaJ and DnaK. The presence of a site in chrI that specifically controls chrII replication suggests a mechanism for communication between the two chromosomes for replication.

## Author Summary

Genome maintenance in dividing cells requires that the chromosomes replicate reliably once per cell cycle, and that this replication be timed to allow for proper segregation of the daughter chromosomes before cell division. In organisms with divided genomes, eukaryotes and a significant class of bacteria, the chromosomes must avoid interference with one another. They exhibit disciplined chromosome choreography, involving several regulators and control circuits that, even in the simplest organisms, are poorly understood. Here we examine the regulatory processes involved in maintaining the two chromosomes of the well-studied and medically important pathogen *Vibrio cholerae*. We provide evidence that a site in chromosome I can control the frequency and timing of replication of chromosome II. The mechanism involves a DNA-mediated remodeling of the chromosome II-specific initiator of replication by the chromosome I site. The site enhances the activity of the protein by differentially affecting its affinity for inhibitory and stimulatory sites on chromosome II. Our results provide the groundwork for determining whether coordination of replication might be a conserved feature that maintains chromosomes in proliferating cells of higher organisms.

## Introduction

Eukaryotes invariably have many chromosomes, whereas one chromosome is the norm in bacteria. Bacteria with more than a single chromosome, although amounting to only about ten percent of known bacterial species, belong to diverse phyla and include important human and plant pathogens [1]. How diverse chromosomes are maintained in individual bacteria is only beginning to be understood.

The bacteria with a multipartite genome have one main chromosome containing most of the housekeeping genes [2]. Replication of this chromosome, which is analogous to the chromosome of monochromosome bacteria, is controlled by the initiator protein, DnaA. DnaA is not only well-conserved in bacteria but also has structural homology to eukaryotic initiators, Cdc6 and Orc proteins [3], [4]. The secondary chromosomes in these bacteria appear to have originated from plasmids. Although plasmids often use DnaA as a replication factor, they do not depend on it to control their copy number; this is accomplished by initiators that they encode themselves. The secondary chromosomes also encode their own initiators to control replication and, like other chromosomes, initiate replication once at a particular time of the cell cycle [2], [5]–[7]. Plasmid replication has no such constraints. Bacteria with multipartite genomes also differ from eukaryotes in that eukaryotic regulators of replication are not chromosome-specific [8].

In all organisms chromosome replication and segregation must be completed prior to cell division. Although the individual chromosomes of bacteria with multipartite genome use different replication and segregation systems, some coordination among the processes that maintain them is to be expected [9]–[11]. We argue that since segregation initiates soon after replication initiation, the coordination is more likely to be at the stage of replication initiation.

Our knowledge of replication control in bacteria comes primarily from the study of the *E. coli* chromosome and plasmids. Multiple modes of control are used, but they all operate primarily through binding of the initiator protein to its sites in the origin of replication, as was originally proposed in the replicon model [12]. It was later found that the initiator also binds outside of the origin and plays important roles in the regulation of replication [13]. In *E. coli*, the initiator, DnaA, has more than 300 near-consensus binding sites distributed over the entire chromosome [14]. As sites are duplicated during replication elongation, their increased number results in titration of free initiators and reduces their availability for binding to the origin, which is important for preventing premature initiation [15]. Subsequent studies showed that some sites play more active roles in controlling replication. For example, the *datA* locus, although originally appeared to be titrating a significant fraction of total DnaA molecules of the cell [16], promotes hydrolysis of DnaA-ATP, the active form of the initiator, to DnaA-ADP, the inactive form [17]. Another mechanism for promoting DnaA-ATP hydrolysis, called RIDA (regulatory inactivation of DnaA), operates during the entire elongation phase as the replication fork passes through the DnaA binding sites [18]. Two other binding sites, called DARS (DnaA reactivating sequence), do the opposite by facilitating the conversion of DnaA-ADP to DnaA-ATP, thus promoting replication [19]. DnaA is also the central mediator of control in other bacteria, but the details of the control can be species specific [20]–[23]. In plasmids, extra binding sites can also be present outside of the origin and they play only inhibitory roles through initiator titration and higher order interactions with the origin sites [6], [24]. Plasmids, being orders of magnitude smaller than the chromosomes, have short elongation periods, and are not known to actively utilize the elongation phase for regulatory purposes.

The importance of initiator binding sites outside of the origin in regulation of chromosomal replication, led us to ask whether plasmid-like secondary chromosomes might also employ such sites for regulation of their replication. Genome-wide distribution of such sites could also provide a mechanism for communication among the individual chromosomes.

Among the bacteria with divided genome, chromosome maintenance has been studied mostly in *Vibrio cholerae*. The bacterium has two circular chromosomes (chrI and chrII) of 2.96 and 1.07 Mb, respectively [25]. They initiate replication at different times and use different initiators, DnaA and RctB for controlling replication of chrI and chrII, respectively [6], [7]. The replication system of chrI appears identical to that of the *E. coli* chromosome, whereas that of chrII is similar to those of plasmids with repeated initiator binding sites (iterons) [9], [24]. The chrII system is more elaborate in that the RctB initiator binds to iterons (12-mers) as well as a second kind of site (39-mers) [26]. The iterons are essential for replication initiation whereas the 39-mers play only inhibitory role that prevents over replication. The iterons antagonize 39-mer activities, thus playing only positive roles. Whether chrII also uses extra RctB binding sites outside the origin, like the DnaA binding sites in other bacterial chromosomes, has not been studied.

Here, we have screened for new RctB binding sites using a genome-wide DNA binding analysis (ChIP-chip). We report the identification of a new region containing additional RctB binding sites (multiple iterons and a 39-mer) in chrII, reminiscent of the *E. coli datA* locus. When provided in multiple copies, these sites were capable of titrating RctB and inhibiting chrII-specific replication. Additionally, a novel RctB binding site was found on chrI. In contrast to the chrII sites, the chrI site enhanced chrII-specific replication, reminiscent of the *E. coli* DARS. These results imply that providing regulatory sites outside the origin could be a general means to exploit the elongation phase for regulating chromosomal replication in bacteria. The chrI site appears to function like a DNA chaperone as it modulates DNA binding of RctB, and by controlling chrII replication, could provide a way to coordinate replication of the two *V. cholerae* chromosomes. The additional layers of chrII control that we reveal here indicate the extent of adaptation required for a (plasmid-like) random mode of replication to become cell cycle regulated.

## Results

### ChrII initiator, RctB, binds outside the chrII replication origin *in vivo*

**Figure 1:**
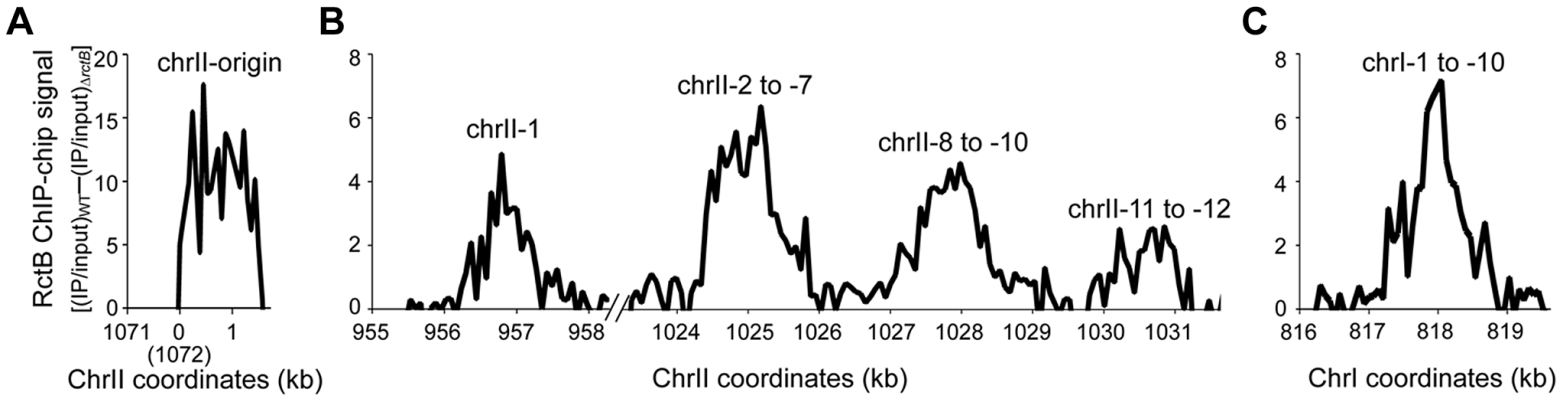
The chrII initiator, RctB, binds to sites outside of the chrII origin. ChIP-chip profiles in the WT *V. cholerae* strain, N16961 (CVC209) in regions where the binding was considered above background. The background was assessed from RctB ChIP signals from Δ*rctB* strain, MCH1 (CVC2099). Regions were selected where the average difference in binding between the two strains was at least 2 in three independent experiments. (The average difference over the entire genome was −0.08±0.86.) The origin region of chrII, where RctB has known binding sites, is shown in (A), regions outside of the origin in chrII are shown in (B), and a region in chrI is shown in (C). chrII-1 to -12 and chrI-1 to -10 refer to fragments from the peak regions of (B) and (C) profiles, respectively, that were tested further for activity.

We analyzed genome-wide binding of RctB in wild type (WT) *V. cholerae* strain N16961 (CVC209) using a chromatin immuno-precipitation and microarray (ChIP-chip) assay. To compensate for the possible heterogeneity in hybridization efficiency of the microarray probes, the hybridized signals from the DNA immunoprecipitated (IP) with RctB antibody was divided by the corresponding signals from the total DNA before immunoprecipitation (input DNA). To avoid non-specific signals, the IP DNA/input DNA values were determined from an *rctB*-deleted strain, MCH1 (CVC2099). MCH1 is a monochromosome mutant of N16961, in which the fusion of chrII to chrI results in deletion of the chrII origin region, including the *rctB* gene [27]. When the difference in IP DNA/input DNA values between the two strains was plotted across the whole genome, a significant enrichment of the IP DNA from the origin region was apparent (Figure 1A). We take this result as validation of the ChIP-chip assay because the origin region is known to have several iterons and 39-mers, the specific binding sites of RctB [26].

A few regions outside of the chrII origin were also enriched. Five regions were selected for which the difference in signal between the WT and MCH1 strains was two or more. Four of the regions were from chrII (Figure 1B) and one from chrI (Figure 1C). The DNA fragments from these regions were tested for their ability to affect RctB-dependent replication *in vivo* and RctB binding to DNA *in vitro*.

### The newly identified RctB binding sites in chrII inhibit mini-chrII replication in *E. coli*

**Figure 2:**
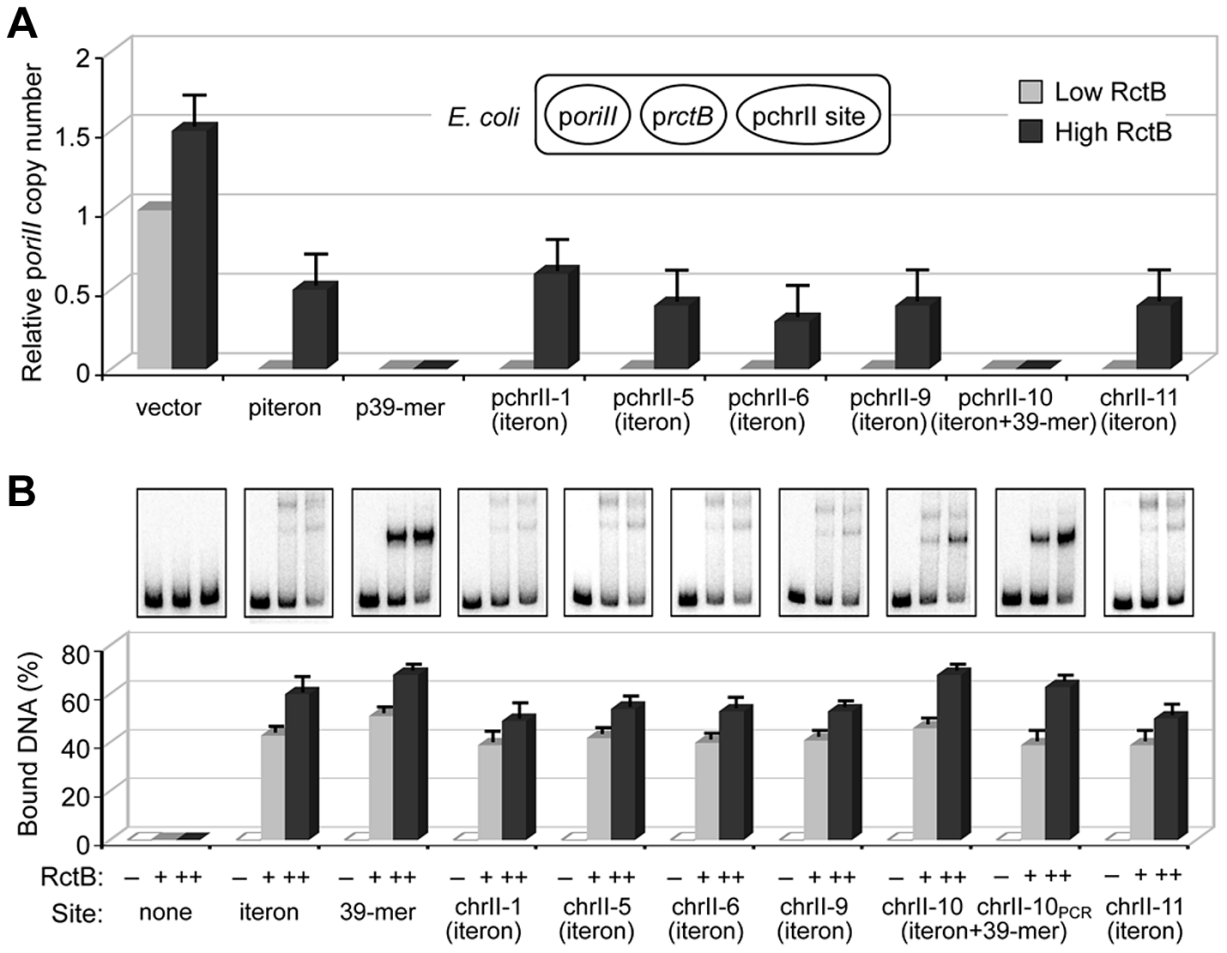
The newly identified chrII sites inhibit mini-chrII replication in *E. coli* and bind purified RctB. (A) The activity of the chrII origin, *oriII*, was tested using a three-plasmid system in *E. coli*, where one plasmid carried *oriII*, p*oriII* (pTVC35), another supplied RctB under arabinose control (pTVC11) and the third was either the empty vector (vector; pTVC243) or carried one of the new sites. As positive controls we used plasmids carried an iteron (piteron; pBH127) or a 39-mer (p39-mer; pTVC222). RctB was supplied at low (light gray bar) and high (dark gray bar) concentrations, using arabinose at 0.002% and 0.2%, respectively. The copy numbers of p*oriII* were normalized to the copy number of p*oriII* (called 1) when the third plasmid was the empty vector (pTVC243) and arabinose was at 0.002%. The mean values and standard deviations are from three independent experiments. (B) Binding of purified RctB to the new sites was tested by EMSA. Percent binding ([intensity of the retarded band/combined intensities of retarded and free bands]×100) at 2 nM (+) or 20 nM (++) are shown by light and dark gray bars, respectively. The error bars are from three independent measurements of band intensities from the same gel.

Sequence analysis of the ChIP-chip peak regions for homology to iterons and 39-mers, revealed homologous sites at 12 places. These putative binding sites were named chrII-1 to chrII-12 (Figure 1B and Table S1). The ability of these sites to influence chrII-specific replication was tested in a three-plasmid system as described [26] (Figure 2A, top). The sites were cloned into a pBR322-derived vector and the resultant plasmids were introduced into *E. coli* with resident p*oriII* and p*rctB* plasmids. The copy number of p*oriII* in the transformants was reduced by six of the sites: chrII-1, -5, -6, -9, -10 and -11, with no significant change by the others (Figure 2A and Table S1). At least one of these six sites is present in each of the four peaks in Figure 1B, which again validated that the ChIP-chip method reported specific binding of RctB. The sites presumably reduced p*oriII* copy number by titrating RctB since increasing the supply of RctB alleviated that reduction (High RctB, Figure 2A and Table S1) [26]. In the case of chrII-10, which has an iteron nested within a 39-mer, increasing the amount of RctB failed to alleviate p*oriII* copy-number reduction. We showed earlier that when the two kinds of site are present in the same fragment, a more potent inhibition of replication results that can not be overcome by increasing RctB [26]. We show below that both the 39-mer and its nested iteron could bind RctB (Figure 2B).

A region centered on the chrII coordinate 1025 kb contains two iterons (the ones present in chrII-5 and -6), several GATC sites and a DnaA box with three mismatches to the consensus sequence, TTATCCACA (Figure S1A). These elements being present in the chrII replication origin motivated us to test whether the region can confer origin function. The cloned fragment did not show origin activity (Figure S1C) (Text S1). The results suggest that the newly recognized chrII sites serve only replication inhibitory activity.

The newly identified sites (five iterons and a 39-mer) that could inhibit chrII replication are present in a span of about 74 kb (coordinates 956828–1,030773), only about 40 kb away from the chrII origin. The locus will thus be duplicated early and be particularly effective in preventing premature initiation by providing additional titration sites. *E. coli datA* is similarly located close to *oriC* at 94.7 min [28].

### The newly identified chrII sites also bind RctB *in vitro*

By electrophoretic mobility shift assay (EMSA), the six chrII sites that could reduce p*oriII* copy number were the only ones proficient in binding RctB *in vitro* (Figure 2B). These sites are also the ones that contained the fully conserved iteron bases, TGATCA (Table S1). The results of ChIP-chip analysis, p*oriII* copy number measurements and EMSA are thus fully consistent. The EMSA pattern of the chrII-10 site appeared as expected for a composite of an iteron and a 39-mer, as the fragment sequence suggested. Since RctB binding to iterons requires the site to be methylated [26], [29], it was possible, using an un-methylated chrII-10 fragment (made by PCR), to test whether its 39-mer could bind RctB on its own. It did (chrII-10_PCR_, Figure 2B). This indicates that the chrII-10 site can function both as an iteron and a 39-mer.

### The newly identified RctB binding site in chrI enhances chrII replication

**Figure 3:**
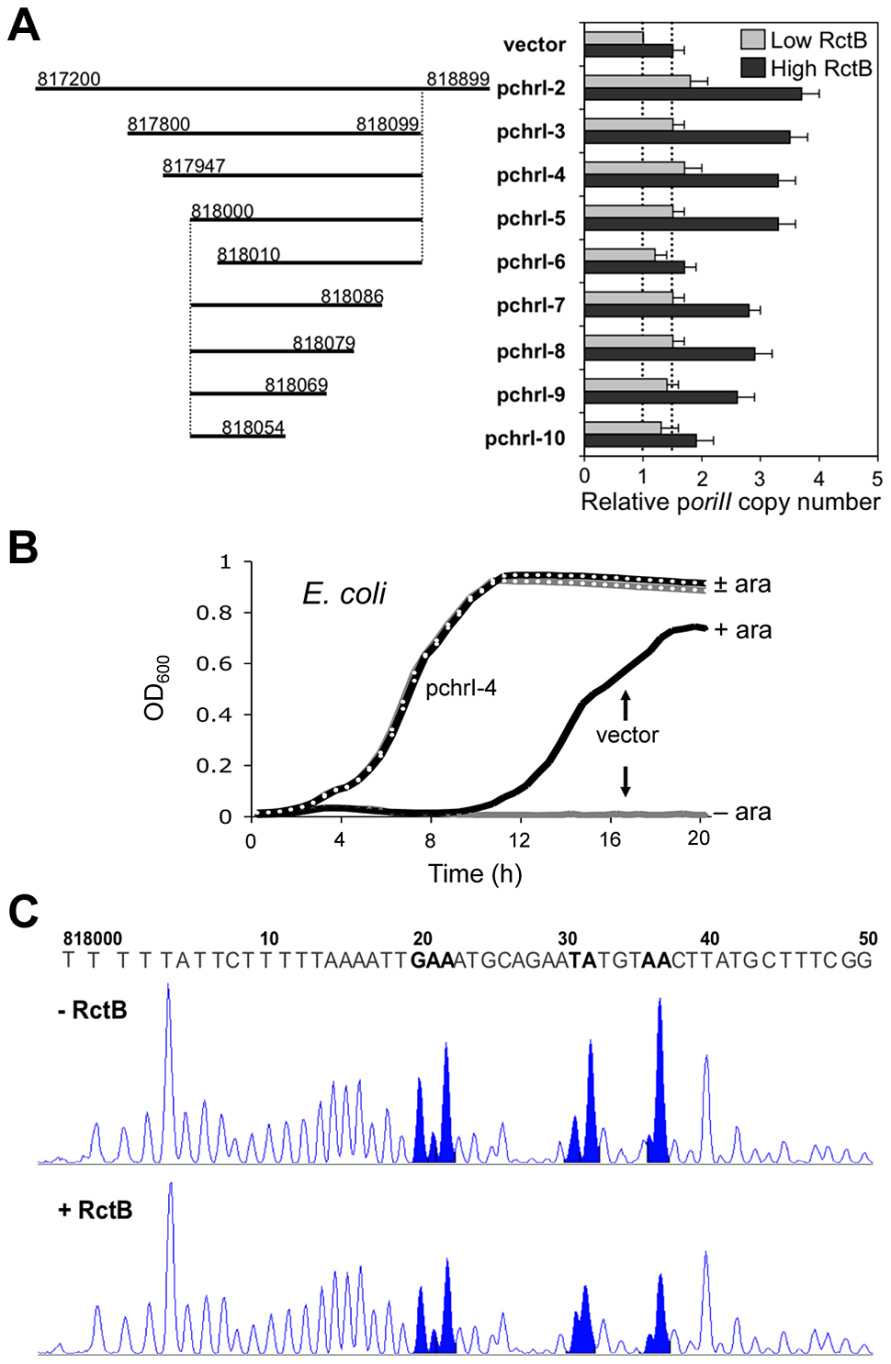
The newly identified chrI site enhances mini-chrII replication, improves cell growth and binds purified RctB. (A) *oriII* activity was tested as in Figure 2A. The copy numbers with the empty vector are shown by dotted lines for reference purposes. (B) Growth of *E. coli* cells harboring three plasmids: p*oriII* (pTVC35), p*rctB* (pTVC11) and either an empty vector (pTVC243; solid lines) or the same vector carrying the chrI-4 fragment (pchrI-4 = pBJH170; dotted lines). The growth was tested in LB under antibiotic selection for all three plasmids, and OD_600_ was monitored using the Synergy HT plate reader (BioTek, USA). Freshly transformed colonies from plates containing 0.2% arabinose were suspended in LB and cultivated at 37°C. (Upon saturation of growth, the cultures were diluted and they grew with their characteristic lag periods, indicating that the lag is unlikely to be due to accumulation of mutants.) The amount of RctB was varied either by omitting the inducer (−ara; gray lines) or by adding the inducer at 0.2% (+ara; black lines). (C) RctB binding to the chrI-4 sequence was tested by DNase I footprinting *in vitro*. The chrI-4 sequence was present in a supercoiled plasmid (pBJH170), and the DNase I treatment was done either in the absence or in the presence of 20 nM RctB. The sites of DNase I cleavage were mapped by primer extension. The extension products are shown only for the first 50 nt of the minimal 70 bp site (as in chrI-9) within which changes (shaded in blue) in the presence of RctB were significant. The DNase I level was adjusted so that there is one nick in the region of interest, making it unlikely that the plasmid was supercoiled during the DNase I probing. It remains possible that only the initial binding of RctB requires supercoiling but once bound the complexes remain stable enough to reveal the footprint in relaxed plasmids.

The region of chrI that by ChIP-chip evidence binds RctB (Figure 1C) contains only one iteron-like sequence with the conserved hexamer, TGATCA. However, a 24 bp fragment containing the hexamer marginally inhibited chrII replication in the three-plasmid assay system used above, suggesting that the hexamer by itself it not a strong RctB binding site (chrI-1; Table S2). When a fragment covering the entire peak region (chrI-2, spanning coordinates 817200–818899) was tested, it significantly increased p*oriII* copy number (Figure 3A). Serial deletion of the fragment from either side (resulting in fragments chrI-3 to -10) showed that the replication-enhancing activity could be narrowed down to 70 bp (as in chrI-9, spanning coordinates 818000–818069). Comparison of chrI-5 and -6 indicates that the leftmost 10 bp sequence of chrI-5 is important for activity. Trimming of the other end had a less dramatic effect (chrI-7 to -10). The 70 bp replication enhancer bore no sequence similarity to either the iterons or the 39-mers. Thus, the enhancer appears to be a new kind of RctB binding site, possibly a 70-mer. Western blotting analysis showed that the level of RctB synthesized in the presence of chrI-4 was comparable to that in the absence of chrI-4 (Figure S3A), indicating that chrI-4 is increasing the activity of RctB and not its concentration.

The replication-enhancing activity of the chrI site was also evident by monitoring the growth of cells dependent on p*oriII* function (Figure 3B). In the three-plasmid system used above, the *rctB* gene in p*rctB* was under the control of an arabinose-inducible promoter, P_BAD_, and when the presence of p*oriII* was selected with appropriate antibiotics, cells grew only in the presence of arabinose. When chrI-4 was present, cells grew in media lacking arabinose but did not increase further upon addition of arabinose. The level of RctB in the absence of arabinose was too low to be detected by Western blotting, indicating that the improved growth by chrI-4 is not due to significant increases in RctB concentration.

Comparison of growth curves of cells with chrI-4, -5 and -6 showed that while cells with chrI-4 and -5 grew similarly, cells with chrI-6 grew similarly to cells with the empty vector (data not shown). Since chrI-6 shows little replication enhancer activity (Figure 3A), these results confirm that the enhanced growth owes to the presence of an active enhancer fragment.

The chrI-4 enhancer fragment contains an AT-rich stretch, several GATC sites and a DnaA box with three mismatches to the consensus sequence (Figure S1B). The fragment, however, failed to show origin function (Figure S1C). It appears that DnaA protein is also not important for the chrI-4 enhancer activity because p*oriII* copy number increased in *dnaA*(ts)204 strain (BR4433) even at the non-permissive temperature of 42°C.

Taken together, these results indicate that a 70-mer site in chrI can significantly enhance the initiator activity of RctB in *E. coli*. Its importance is further suggested by its conservation in the *Vibrio* genus (Figure S4).

### The chrI site that enhances chrII replication requires supercoiling to bind RctB *in vitro*

Here we asked whether the enhancer site could bind purified RctB *in vitro*, as would be expected from the ChIP-chip results. When the chrI-4 DNA was used as a linear fragment, no binding could be detected by EMSA (data not shown). Linear fragments of chrI-4 DNA also failed to inhibit RctB binding to a 39-mer site (data not shown). The effect on RctB binding to iterons was ambiguous because the binding was weak to start with [30]. However, when the chrI-4 sequence was present in a supercoiled plasmid (pBJH170), although we could not detect RctB binding to the plasmid itself by EMSA (Figure S5A), the plasmid caused a reduction in RctB binding to a 39-mer (Figure S5B). These results indicate that RctB can interact with the chrI site only in the supercoiled form. This conclusion was supported by DNase I footprinting of RctB, where a footprint could be detected only when the chrI-4 sequence was in a supercoiled plasmid (Figure 3C).

The bases protected from DNase I cleavage were distributed within a stretch of 18 bp (coordinates 818020–818037) in the middle of the 70-mer site. When the specific protected bases (shown in bold here and in Figure S6A) were mutated from **GAA**ATGCAGAA**TA**TGT**AA**C to **AGG**ATGCAGAA**CG**TGT**GG**C, RctB essentially failed to protect the mutant site (chrI-9m) from DNase I cleavage. The chrI-9m also lost its replication enhancer activity (Figure S6B). As will be discussed later, extra copies of the enhancer site decreases growth of *V. cholera*. The growth inhibition was not seen when the chrI-9m was used instead of chrI-9 (Figure S6C). These results indicate that RctB directly interacts with the replication enhancer site in chrI.

### The replication enhancer site in chrI increases RctB binding to iterons and decreases RctB binding to 39-mer in *E. coli*

**Figure 4:**
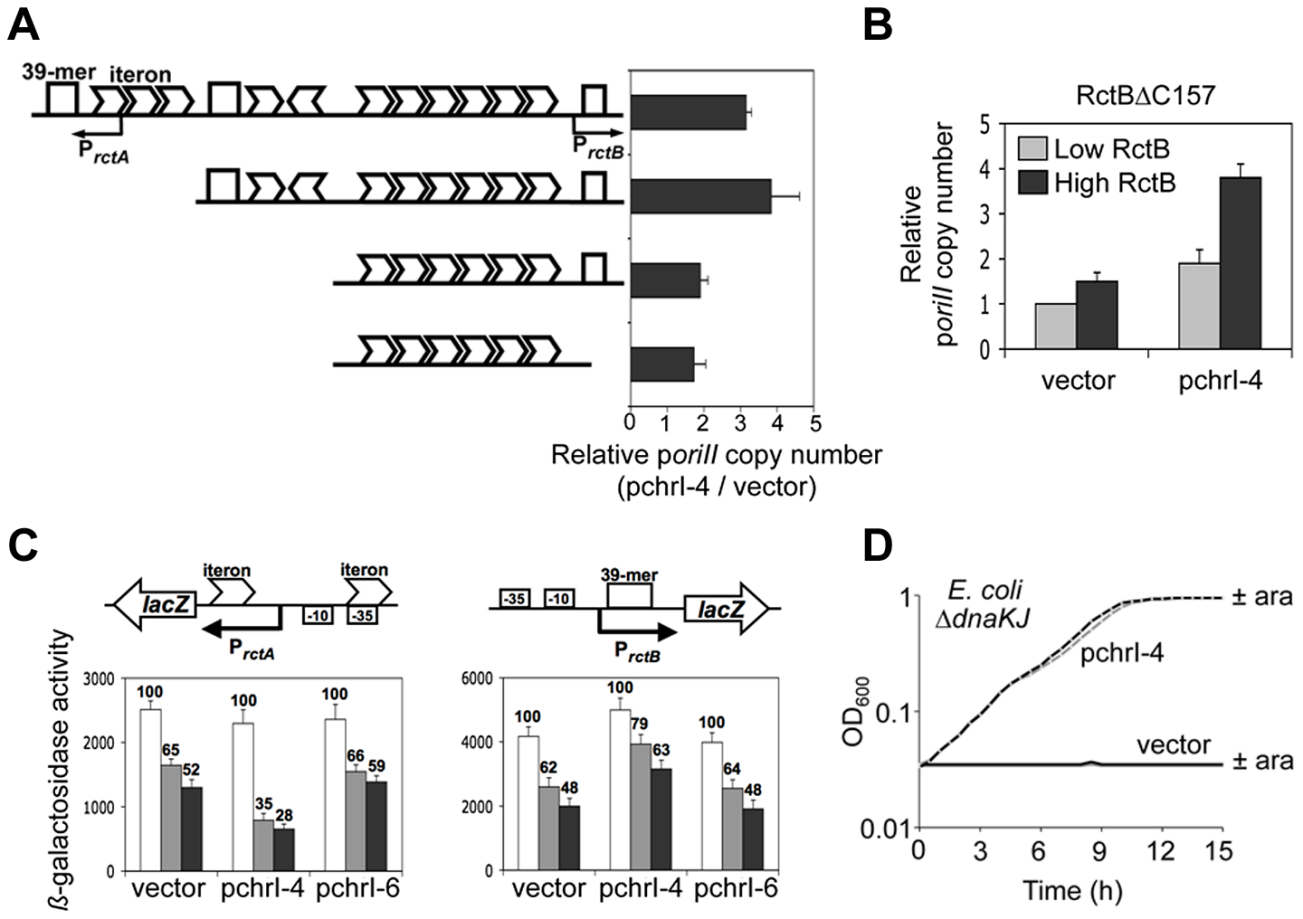
The chrI site modulates DNA binding of RctB and enhances p*oriII* activity in Δ*dnaKJ* host. (A) The effect of chrI-4 (present in pBJH170) on *oriII* activity was tested as in Figure 2A, except that four different the p*oriII* plasmids were used (from top pTVC210, pTVC25, pTVC31 and pTVC524), containing different numbers of regulatory iterons and 39-mer sites (arrowheads and squares, respectively). Copy numbers were determined at high RctB concentration (0.2% arabinose) only. At low RctB concentration the copy number 39-mer carrying plasmids, pTVC210 and pTVC25, were too low to be measured reliably. (B) Non-essentiality of the 39-mer binding activity of RctB in the enhancer function. The copy numbers were measured as in (A) except that p*oriII* was pTVC210 and RctB was a 39-mer binding defective mutant (RctBΔC157) [30]. (C) *In vivo* binding of RctB to iterons and a 39-mer in the presence of empty vector (pKT25) or the same vector carrying chrI-4 or chrI-6 (pBJH188 or pBJH243, respectively). The vector and pchrI-6 were used as negative controls. The chrII origin contains two promoters, P*_rctA_* and P*_rctB_*, with overlapping iterons and a 39-mer, respectively. RctB binding to these sites represses the promoters. Activities of the promoters (in pTVC126 and pTVC500, respectively) were determined at three concentrations of arabinose at 0% (white bars), 0.002% (gray bars) and 0.2% (black bars). The error bars are from three independent measurements. (D) The effect of chrI-4 on the growth of Δ*dnaKJ* cells (BR4392) carrying p*oriII* (pTVC31), p*rctB* (pTVC11) and either the empty vector (pTVC243) (solid lines) or the same vector containing chrI-4 (pBJH170) (dotted lines). The cells were initially grown in LB containing antibiotics to select all three plasmids and 0.2% arabinose. At time zero, the cultures were diluted 1000× with fresh medium containing antibiotics and either no arabinose (gray lines) or 0.002% arabinose (black line).

Since the chrI site enhances replication through interactions with RctB without affecting RctB concentration, the mechanism of enhancement is likely to be by altering the regulatory activity of the initiator. To begin to understand the mechanism, we tested the effect of the replication enhancer on the activity of p*oriII* plasmids that have different copy numbers because of differing numbers of iterons and 39-mers they carry (Figure 4A). If the mechanism of replication enhancement is by lowering the inhibitory activity, then a copy number increase is expected to be significant only in 39-mer carrying p*oriII* plasmids, since 39-mers are the primary inhibitors of chrII replication. However, the presence of chrI-4 *in trans* increased copy number of p*oriII* whether or not the 39-mers were present in p*oriII* plasmids, although the increase was more pronounced in 39-mer carrying plasmids. Replication enhancement was also seen with the RctB mutant, ΔC157, which is defective in 39-mer binding [30] (Figure 4B). From what follows, it appears that the enhancer can promote replication directly by promoting iteron binding of RctB and indirectly by reducing 39-mer binding of RctB.

To assay the effect of the enhancer on RctB binding to iterons and 39-mers, we took advantage of the presence of two natural promoters in the origin of chrII. One promoter, P*_rctA_*, is associated with two iterons, and the other promoter, P*_rctB_*, with an operator that is naturally a truncated 39-mer (a 29-mer) but can also be replaced with a 39-mer without changing the operator activity [30]. The promoter activities were measured after fusing them to the *lacZ* reporter gene (of a multicopy plasmid, pMLB1109). RctB represses the activity of both the promoters, and this repression has been utilized as a convenient proxy for RctB binding to its specific sites *in vivo* [30]–[32]. When the chrI-4 site was present, RctB could more effectively repress P*_rctA_* (the promoter activity decreased from 65±4 to 35±5% at low RctB concentration), suggesting that chrI-4 improves RctB binding to iterons (Figure 4C). In the case of P*_rctB_*, chrI-4 had the opposite effect; here the promoter repression was less in the presence of chrI-4 (the promoter activity increased from 62±7 to 79±6% at low RctB concentration), suggesting that chrI-4 makes 39-mer binding less effective. The decreased binding of RctB to a 39-mer in the presence of chrI-4 was also seen *in vitro* (Figure S5B). When chrI-4 was replaced with chrI-6, which is defective in enhancer activity, repression for both the promoters did not change significantly from those seen with the empty vector. Since 39-mers inhibit replication and iterons activate replication [26], reduced binding to the former and increased binding to the latter are both consistent with the observed replication enhancement by chrI-4. Together, these results suggest that the enhancer remodels RctB to alter its DNA binding activities.

### The enhancer site in chrI possesses chaperone-like activity on RctB

RctB binding to both iterons and 39-mers are greatly stimulated in the presence of DnaJ and DnaK chaperones *in vitro* and in *E. coli* [30]. As shown in Figure 4D, when the presence of p*oriII* was selected in an *E. coli* Δ*dnaKJ* host (BR4392), the cells growth was negligible whether RctB was supplied at basal or induced levels (using 0.002% arabinose). In contrast, even the basal level of RctB allowed near maximal growth in the presence of chrI-4. We confirmed that the increased growth was due to increased *oriII* activity; the copy number of p*oriII*, measured by qPCR, increased 3.1±0.5 fold in the presence of chrI-4. The induced level of RctB used in these experiments was undetectable by Western analysis. Using quantitative RT-PCR, we confirmed that upon *rctB* induction, the gene was expressed at similar levels in cells with and without chrI-4 (8.2±2.1 and 7.6±1.9, respectively). When induced with 0.2% arabinose, RctB could be detected by Western analysis and its level was similar in cells with and without chrI-4 (Figure S3A, lanes 7,8). The results support the earlier inference that the enhancer is increasing the activity of RctB and not its concentration.

The altered binding of RctB to iterons and to a 39-mer in the presence of chrI-4 was also seen in a *dnaK7* strain (BR4390) (Figure S7). These results suggest that chrI-4 could be functioning like a DNA chaperone to remodel RctB. The remodeling, however, appears to be different in the two cases because the chaperones improve binding to both iterons and the 39-mer (tested *in vitro*; [30]), whereas chrI-4 improved binding only to the iterons but decreased binding to the 39-mer. We note that RctB binding to chrI-4 could be seen *in vitro* only in the presence of chaperones (Figure 3C, Figures S5B and S6A). It appears that *in vitro*, chrI-4 remodels RctB after it has been acted upon by chaperones. The apparent *dnaKJ* independence could be due to the presence of other chaperones that substitute for DnaJ and DnaK activities. Alternatively, the purification of RctB from overproducing cells could have affected its activity, imparting to it a form different from that present in a Δ*dnaKJ* or *dnaK7* host.

### The chrII sites reduce and the chrI site enhances chrII-specific replication in *V. cholerae*

**Figure 5:**
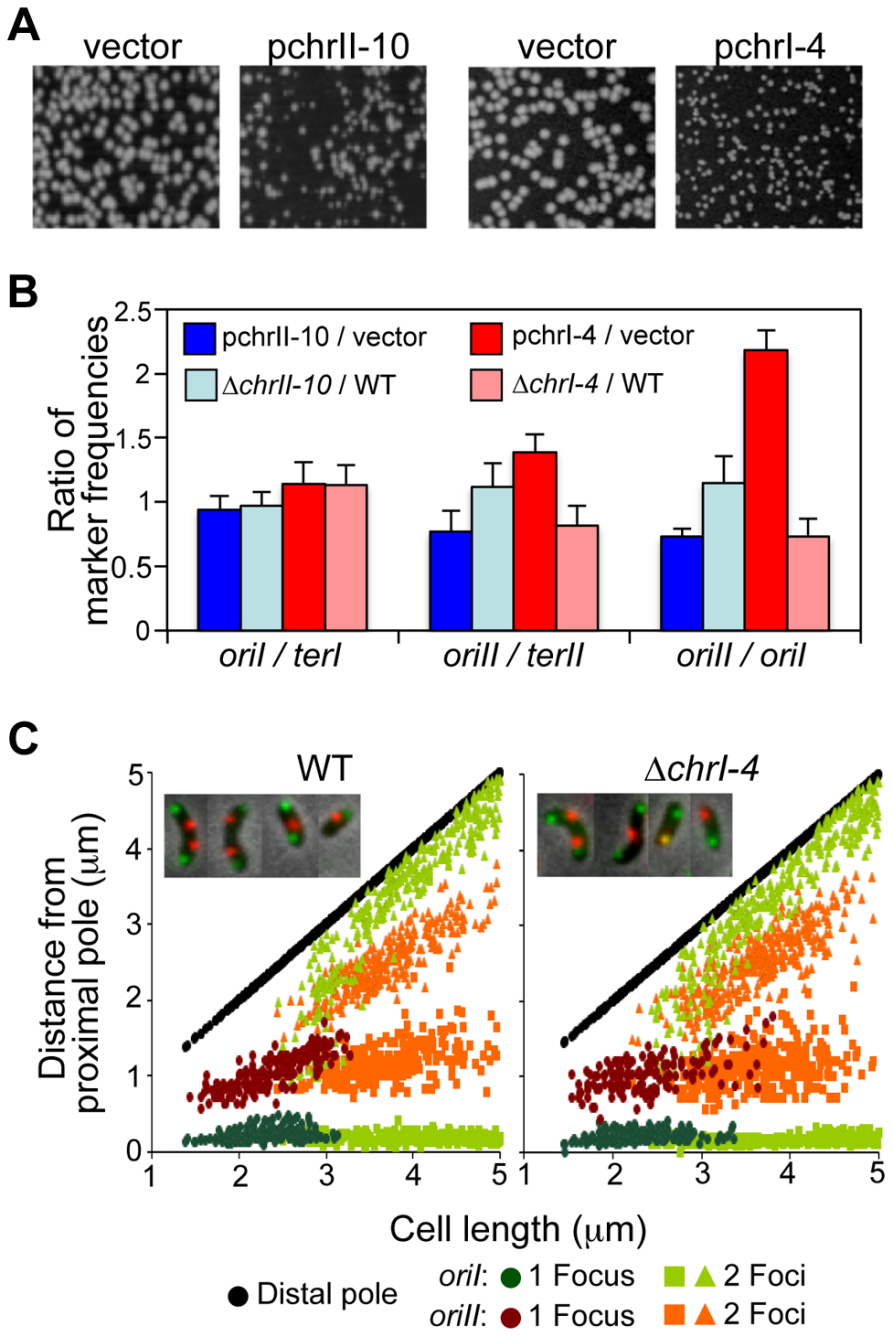
The newly identified RctB binding sites affect *V. cholerae* growth and chromosomal replication. (A) Growth was monitored by colony size of WT *V. cholerae* cells (CVC209) transformed with either an empty vector (pBR322 derivative, pTVC243) or the same vector containing chrII-10 (pTVC350) (first two panels), or an empty vector of lower copy number (pACYC177) and the same vector containing chrI-4 (pBJH188) (last two panels). (B) Chromosomal replication was tested by measuring relative frequencies of four markers: *oriI*, *oriII*, *terI* and *terII*, in cells with pchrII-10 (blue bars) or pchrI-4 (red bars). The relative frequencies were then expressed after dividing the corresponding relative frequencies in cells with empty vectors. Plasmids used were as in (A). Relative marker frequencies were also determined for the Δ*chrII-10* mutant (CVC2565) and Δ*chrI-4* mutant (CVC2542), and expressed after dividing with the corresponding ratios from the otherwise isogenic WT cell (CVC1121) (light blue and red bars). Data are averages from three independent experiments. (C) Localization of *oriI* and *oriII* in WT (CVC2553) and Δ*chrI-4* mutant (CVC2554) strains. *oriI* and *oriII* were marked by inserting P1*parS* and pMT*parS*, and detected by GFP-P1ParB and mCherry-pMTParB, respectively. Plots show focus positions in cells with one focus (dark green for *oriI* and dark orange for *oriII*) and two foci (light green for *oriI* or light orange for *oriII*). Focus positions were measured from the pole from which the nearest focus was closer than the nearest focus from the opposite pole. The pole used to measure focus positions is placed on the abscissa and the other (distal) pole is shown as black circles. 500 cells were analyzed in each experiment.

To test whether the newly identified RctB binding sites have a replication phenotype in the native host (*V. cholerae*), we chose the chrII-10 site, which contains both an iteron and a 39-mer and showed the strongest replication inhibitory activity, and the chrI-4 site, which showed the maximal replication enhancer activity. Plasmids carrying these sites were used to transform WT *V. cholerae* cells (CVC209). The colony size of the transformants was significantly smaller in the presence of either chrII-10 or chrI-4 (Figure 5A). The reduction in the case of chrII-10 was expected because the iterons and 39-mers are known to titrate RctB, which could reduce *V. cholerae* growth by reducing chrII replication [26]. In fact, when additional iterons were added to the chrII-10 plasmid, no viable transformants were recovered. However, deletion of the chrII-10 site alone from the *V. cholerae* chromosome did not affect viability and did not show a growth phenotype (Figure S2A).

The reduction of colony size in the case of chrI-4 is also expected due to over-activity of *oriII*. Increased chrII copy number due to overexpression of RctB or due to a copy-up *rctB* mutation reduced cell growth previously [33], [34]. That over activity of *oriII* could be the cause of growth reduction was confirmed by measuring *oriI/terI*, *oriII/terII*, and *oriII/oriI* marker ratios using qPCR (Figure 5B). The difference in *oriI/terI* ratios in cells with and without chrII-10 or chrI-4 sites was insignificant, suggesting that these newly identified RctB sites do not affect initiation of chrI replication. As expected, both chrII-10 and chrI-4 oppositely affected chrII replication: In the presence of extra copies of chrII-10, the *oriII/terII* ratio decreased, and in the presence of chrI-4, the ratio increased (pchrII-10/vector and pchrI-4/vector, Figure 5B). Deletion of the sites showed the opposite effect, also as expected (Δ*chrII*-*10*/WT and Δ*chrI*-*4/*WT, Figure 5B). These results are consistent with the inference from p*oriII* copy number measurements in *E. coli* that indicated that the chrII sites inhibit and the chrI site enhances *oriII*-specific replication.

### The enhancer activity is physiologically relevant

The chrI-4 site could be deleted without causing a significant growth change in *V. cholerae* (WT/vector vs. Δ*chrI*-*4/*vector, Figure S2B). When the *chrI-4* deleted host was transformed with a medium copy number plasmid carrying the chrI-4 sequence, pchrI-4 (pBJH188, pACYC177-derived), there was no significant reduction in the growth of the transformants either (Δ*chrI*-*4/*pchrI-4). The same plasmid however reduced the growth of the WT *V. cholerae* host (WT/pchrI-4). Thus, a change of chrI-4 copy number by one (that exists between the WT and the chrI-4 deleted hosts) can cause a detectable growth phenotype in the presence of pchrI-4.

Although the chrI-4 deleted host did not show a growth phenotype, a reduction in *oriII* copy number compared to that in WT was evident by marker frequency analysis using qPCR (Δ*chrI-4*/WT, Figure 5B). These results indicate that the activity of the chrI-4 site although modest is physiologically relevant.

*E. coli* deleted for regulators like SeqA does not show a strong growth phenotype but replication initiation timing becomes heterogeneous in such cells [35]. When duplication timing of the *oriI* and *oriII* loci were determined by fluorescence microscopy, it was delayed in Δ*chrI-4* cells specifically for *oriII* (Figure 5C). Normally, the origins duplicate (and segregate) at a particular cell length. Cells with one *oriII* focus could be longer in Δ*chrI-4* background compared to such cells in the WT. The modal cell length also increased and the separation between the duplicated foci became heterogeneous upon chrI-4 deletion (Figure S8A and B, respectively). ChrII-less cells that are frequently found in Δ*parAB2* mutants were not found in Δ*chrI-4* mutants [36] (Figure S2C). These results suggest that the chrI-4 site normally functions in controlling the timing of chrII replication initiation.

## Discussion

Here we show that RctB, the specific replication initiator for *V. cholerae* chrII, has binding sites outside of the origin region of chrII. Five sites were found in chrII and one in chrI. The chrII sites are homologous to the sites present in the origin and are capable of inhibiting chrII replication initiation, whereas the site on chrI, which is a new kind of site, plays the opposite role of enhancing initiation. The inhibition most likely involves titration of the initiator, whereas the enhancement is likely due to remodeling of the initiator. Unlike the many *trans*-acting factors (including DnaA, IHF and Dam) encoded in chrI that participate in the replication of both chromosomes, the new RctB binding site in chrI appears dedicated to chrII replication. We propose that the site may contribute to coordinate replication timing of the two chromosomes, as discussed below.

### Communication between chromosomes of *V. cholerae*

Communication among chromosomes is an open question in bacteria with divided genomes. In *V. cholerae*, the possibility of communication was suggested by the finding that replication of both chrI and chrII involves common factors such as DnaA and Dam [9]. To the extent studied, these factors appear to be unlikely candidates for coordinating replication, since DnaA is not a regulator of chrII replication and Dam is essential only for chrII replication [7], [27], [29]. Moreover, blocking chrII replication specifically did not disturb the timing and initiation rate of chrI [37]. The influence of chrI replication on chrII replication was heretofore unknown.

As discussed in the Introduction, DnaA binding sites outside of the origin serve as major regulators of *E. coli* chromosomal replication. This motivated us to search for extra binding sites of RctB outside the chrII origin region. In iterons of chrII, only the hexamer TGATCA is fully conserved. The hexamer includes the Dam methylation site, GATC, and methylation at the adenine residue is required for efficient RctB binding and chrII replication [27], [29]. The importance of the less conserved flanking sequences of the hexamers has not been tested. If the hexamer were to be sufficient for RctB binding, then the occurrence of about 1000 copies of such hexamers genome-wide would suggest that RctB titration to those sites could have a major regulatory consequence to chrII replication initiation, analogous to how the genome-wide presence of DnaA binding sites contribute to *E. coli* replication control. ChrI, being three times the size of chrII, could then play a proportionately larger role in controlling chrII replication. However, the TGATCA is not sufficient for RctB binding (foot note ‘a’, Table S2) and ChIP-chip experiments revealed only five titration sites and they were all in chrII. ChrI thus cannot be regulating chrII replication by titrating RctB.

All the five titration-sites being located close to the origin duplicate shortly after chrII replication initiation. The early increase in the number of sites would maximize initiator titration and, like several previously described mechanisms, could contribute to prevention of premature reinitiation of chrII replication. This role being reflexive, can serve only indirectly in inter-chromosome coordination. The implication is that the intrinsic chrII replication control system needs to be efficient for an external signal from chrI to be effective in precisely timing the replication of the two chromosomes. By contributing to the overall replication control of chrII, the five sites could also be contributing indirectly to coordinate replication initiation. So far, we have deleted only the chrII-10 site without significant effect on *oriII* activity (Figure S2A). A possible reason could be that the site is one of 22 regulatory sites of chrII: 16 iterons (11 in the origin and five identified in this work, Table S1), four 39-mers (three in the origin and one identified in this work, Table S1), one *parS2-B* [38] and the chrI enhancer, and the deletion of one out of 22 sites may not be expected to have a strong effect.

ChrI also contains one of the *parS2* sites that bind the chrII-specific segregation protein ParB2 [39]. The functional significance of this finding has not been determined and it is not clear whether the two chromosomes communicate for segregation. Both chromosomes also encode a dimer resolution system that monomerizes chromosomal dimers that can form by homologous recombination between replicated sisters [40]. Dimer resolution before cell division is required for proper segregation of sister chromosomes. Although in *V. cholerae* the resolution sites (*dif*) are chromosome-specific, the same set of proteins (XerC, XerD and FtsK) act on those sites and they are all encoded in chrI. It is possible that at the time of cell division the final stages of segregation of the two chromosomes are coordinated through these proteins. However, there is no evidence that completion of segregation dictates the timing of replication initiation.

### Applicability to chrII of principles that govern replication of the *E. coli* chromosome

Whereas plasmids generally initiate replication throughout the cell cycle [41], chrII despite its presumed plasmid origin initiates replication at a particular time of the cell cycle, like other chromosomes [6]. The chrII replication system appears to have retained not only the characteristics of the replication system of its presumed progenitor-plasmid, but also added several, if not all, mechanisms used by the *E. coli* chromosome to time its replication initiation, as detailed below.

The *E. coli* chromosome origin has abundant Dam methylation sites at which Dam and SeqA proteins act, and extra initiator (DnaA) titration sites outside of the origin, including special DnaA binding sites called *datA* and DARS that inhibit and activate replication, respectively [17], [19]. The chrII origin is also equally enriched for Dam methylation sites and depends upon Dam and SeqA for replication initiation and its control. As we report here, extra titration sites are present in chrII. The 39-mers, which are special replication inhibitory sites of chrII not found in plasmids, can be thought of as functional equivalents of *datA*. Similarly, the enhancer of chrII replication present in chrI can be likened to DARS. The extra features of chromosomal replication might have been necessary to accomplish once per cell cycle replication and proper timing of replication initiation, which are apparently less critical requirements for plasmid replication. One requirement for the tighter control of chrII replication versus plasmid replication appears to be prevention of over-replication of chrII, which is detrimental to *V. cholerae* growth ([33]; this study). It seems also possible that the deleterious effect of extra copies of chrI-4 is not due to the extra copies of chrII *per se*, but rather to improperly timed initiation of chrII (Figure 5C) such that simultaneous termination of the two chromosomes is compromised and some sort of partitioning problem arises.

### The chaperone-like nature of the chrI site

Here we find that the chrI enhancer site increases RctB binding to iterons, and decreases RctB binding to a 39-mer. The increase in iteron binding can promote replication initiation directly and the decrease in 39-mer binding can do so indirectly. The two activities could be seen as independent; the presence of a 39-mer was not required to see the stimulation of iteron binding, and vice versa (Figure 4C). Replication stimulation activity was also seen using an *oriII* plasmid devoid of 39-mers (Figure 4A) and using an RctB mutant, ΔC157, which is defective in 39-mer binding [30] (Figure 4B). It is possible that the chrI site remodels RctB in such a way that it alters its binding to both the iteron and the 39-mer.

As was reported earlier, we find that the absence of the chaperones DnaJ and DnaK greatly inhibited the growth of cells that are dependent on the functioning of the chrII origin [30]. This inhibition was relieved when the chrI site was provided *in trans* in a Δ*danKJ* host (Figure 4D). These results can be understood if the enhancer promotes RctB activity by remodeling the protein in the absence of DnaJ and DnaK. It is common among DNA binding proteins to change their conformation upon interaction with their cognate DNA binding sites [17], [19], [42]–[45].

The remodeling activity of the enhancer was also suggested by determination of the RctB level in *V. cholerae*. Upon deletion of the chrI-4 site, the initiator level increased by 58±24% compared to that in the WT cell (Figure S3B). There was also increase in *rctB* transcription, although the increase was less significant: the ratio of *rctB* transcripts in Δ*chrI-4*/WT was 1.28±0.34 by qRT-PCR and 1.23±0.22 by promoter fusion to *lacZ* (Figure S3C). The altered expression of *rctB* is expected considering that it is an autorepressed gene and chrI-4 site alters RctB DNA binding (Figure 4C) [31], [46]. Since in Δ*chrI-4* cells chrII replication tends to decrease (Figure 5B), the results suggest that, in spite of the increase in RctB concentration, the initiator activity can decrease in the absence of chrI-4 site. In the presence of pchrI-4, however, there was no significant increase in RctB concentration either in *V. cholerae* or in *E. coli* (Figure S3A and B). Together, the results suggest that changing the initiator activity is the main role of the chrI-4 site.

The *E. coli* sites *datA* and DARS that inhibit and activate replication, respectively, possibly also remodel DnaA. The initiator DnaA is an ATPase and to serve as initiator DnaA needs to be bound to ATP. The *datA* site inhibits initiation by converting DnaA-ATP to DnaA-ADP and the DARS sites promote initiation by doing the opposite conversion of DnaA-ADP to DnaA-ATP [17], [19]. These conversions probably require structural changes in DnaA to increase the ATPase activity and release the bound ADP, respectively. The *datA* and DARS sites may be remodeling the cognate initiator like the chrI enhancer site.

Like DnaA, RctB has been reported to be a weak ATPase [34]. In the same study, ATP was found to strongly inhibit RctB binding to the chrII origin DNA, although in our hands ATP showed only a nominal inhibitory effect (Figure S9A, lanes 2 and 6). On the contrary, ATP was required to promote the robust stimulatory activity of the chaperones on RctB binding to 39-mers (lanes 10 and 12). In the presence of chaperones, which are naturally present *in vivo*, ATP stimulated binding even in the presence of vast excess of ATP (5 mM as opposed to the optimal concentration of 0.1 mM). These results indicate that the chaperones override any inhibitory activity of ATP. In the absence of chaperones, the nominal inhibitory activity of ATP on DNA binding of RctB was also not affected significantly in the presence of the chrII-10 or the chrI-4 site (Figure S9B). In the presence of these sites, the ATPase activity of RctB also did not change significantly (Figure S9C). We also tested a mutant RctB (R269S) whose DNA-binding was reported not to be inhibited by ATP [34]. The mutant, as reported, conferred a higher copy number to a mini-chrII plasmid in *E. coli* (Figure S9D). The mutant however remained sensitive to replication inhibition by the chrII-10 site and replication enhancement by the chrI-4 site. Together the results suggest that the enhancer is unlikely to be functioning by altering the ATPase activity of RctB.

In iteron-based plasmids, dissociation of initiator dimers is the main mechanism by which chaperone proteins stimulate replication [47]–[49]. RctB binds to iterons both as monomer and dimer. The monomer binding is thought to be conducive to replication initiation since copy-up mutants of RctB were more proficient in binding as monomer [30], [50]. However, using a bacterial two-hybrid system and a second assay based on λ*P_R_* repression by the λ repressor [51], [52], the chrI-4 site did not appear to affect RctB dimerization (Figure S10A and B, respectively). The mechanism of enhancement appears unlikely to be by reduction of RctB dimerization.

Remodeling of RctB that leads to increased iteron binding and decreased 39-mer binding appears to be the primary mechanism of the chrI-4 enhancer function.

### The enhancer structure and function

The chrI site is a new kind of RctB binding site, as it has no homology to the sites present in the chrII origin. The site size is also large, ∼70 bp, whereas typical protein binding sites in bacteria are seldom even half that long. The requirements for an AT rich stretch and supercoiling suggest that the site needs to be in the single-stranded state to be active. We tried RctB binding individually to two complimentary single strands of the chrI site (as 70-mer oligonucleotides) by EMSA but without success (data not shown). Both the strands are apparently required for the site to assume proper conformation driven by supercoiling. We considered whether transcriptional activity of the site could effect this change. A promoter is present within the minimal sequence required for enhancer activity, chrI-9, spanning coordinates 818000–818069 (Figure S11A). This promoter was not active in a longer fragment, chrI-4. The extra sequences (817947–818000) present in chrI-4 upstream of chrI-9 apparently repress the promoter by a mechanism that remains to be explored. In any event, when the promoter region was mutated at the −35 (m1) or −10 (m2), or at both sites in chrI-4 and chrI-9, the promoter activity decreased in m2 and in the double mutant, but the replication enhancer activity was not significantly affected (Figure S11B and C). These results indicate that the promoter activity internal to the chrI site is not critical for the replication enhancer activity.

Passage of the replication fork across the chrI site might also cause the conformational switch to the single-stranded state. Moreover, by the passage of the fork the enhancer site concentration would double, which should increase the fraction of remodeled RctB and thus help time the chrII replication initiation more effectively. About 55% of the 2.97 Mb chrI will have duplicated before a replication fork reaches the replication enhancer site it carries. Since the two chromosomes terminate at about the same time [19], by the time chrII replication starts, the chrI replication have proceeded even further by about 10% on each replichore, which would allow ample opportunity for RctB to interact with the nascent enhancers. It is tempting to speculate that the duplication of the replication enhancer site in chrI shortly before the time at which chrII must initiate if the two chromosomes are to terminate simultaneously, is important for this coordination of replication of the two chromosomes.

RctB-enhancer interaction possibly stimulates chrII replication in one of two ways. The enhancer-bound RctB might directly engage with the initiation complex at *oriII*, so as to stimulate replication, analogous to the functioning of *cis*-acting transcriptional enhancers in bacteria and eukaryotes. The interactions can also happen *in trans* between chromosomes as in “chromosome kissing” [53], [54]. However, so far we have failed to detect direct engagement of the enhancer with the initiation complex by the chromosome conformation capture (3C) assay. Alternatively, interactions with the enhancer might lead to a conformational change, which is stable enough for the remodeled initiator to reach the chrII origin by diffusion.

In addition to *E. coli* DARS, replication enhancer sites have been found in plasmids pT181 and pSC101 [55], [56]. In both cases, the enhancer sites, like the chrI enhancer site, bear no similarity to initiator binding sites in the origin. In pSC101, the enhancer has gyrase binding sites, which promote replication by increasing the negative superhelicity of the plasmid, particularly near the replication origin, that favors initiator-origin interaction. The *cmp* locus of pT181 is also believed to promote initiator-origin interaction, but the mechanism is not known. On the other hand, DARS and *datA* both have recognizable DnaA binding sites, which are essential for their function. A deeper mechanistic understanding of the chrII replication enhancer function will require more structural probing of RctB and its likely alternation in presence of the enhancer.

## Materials and Methods

### Strains and plasmids

*E. coli* and *V. cholerae* strains, and plasmids used in this study are listed in Table S3.

### ChIP-chip assay

ChIP-chip assay was performed exactly as described [38]. Cells used here were *V. cholerae* WT (CVC209) and MCH1 (CVC2099). As before, ChIP (Cy5) signals were divided by corresponding input DNA (Cy3) from three independent experiments. The difference in mean Cy5/Cy3 signals between the WT and MCH1 strains was plotted in Figure 1.

### Plasmid copy number measurement

The copy number of p*oriII* was measured using a three-plasmid system as described [26]. *E. coli* (BR8706) cells harboring the p*oriII* (pTVC25, 31, 35, 210 or 524) and *prctB* (pTVC11) that supplies the initiator from an arabinose inducible promoter, was transformed with a pBR322-derived plasmid containing one of the newly identified RctB binding sites. The plasmid copy number was determined by harvesting overnight grown cells directly from transformation plates containing either 0.002 or 0.2% arabinose, the conditions referred to in the text as supplying RctB at low or high levels, respectively.

### EMSA

EMSA was performed as described [57]. A total of 1.2 pmol of DNA fragment, carrying one copy of a binding site with 100 bp of vector sequences (pTVC243) at both flanks, was end-labeled with ^32^P and reacted with RctB at two different concentrations (2 and 20 nM). The binding was monitored using a 5% polyacrylamide gel and autoradiography. For competitive binding assay (Figure S4), DNA was not radiolabeled and visualized after staining with SYBR Green EMSA nucleic acid gel stain at 10,000× dilution for 30 min at room temperature (Molecular Probes).

### DNase I footprinting assay

The binding of RctB was performed under the same condition as was used for EMSA. The binding reactions were treated with 0.01 unit of DNase I (Promega) for 1 min at room temperature. DNaseI was inactivated by adding 15 mM EDTA followed by heating (95°C, 10 min). The DNA was purified using Qiaquick PCR purification kit (Qiagen). Primer extension was performed using 6 FAM-labeled primer and Thermo Sequenase polymerase (USB) according to the manufacturer's protocol. Amplified products were purified using Qiaquick PCR purification kit. The purified products along with GeneScan 500-ROX size standard (Applied Biosystems) were denatured by adding Hi-Di formamide (Applied Biosystems) followed by heating (75°C, 10 min) and immediately chilled on ice. The single stranded DNA was analyzed on an ABI 3130×l Genetic Analyzer (Applied Biosystems) and data was analyzed using GeneMapper (v. 3.7) software.

### Deletion of *chrII-10* and *chrI-4* sequences from *V. cholerae*

The deletion was achieved in two steps. First, the chrI-4 sequence was substituted with an FRT-Zeo-FRT cassette by the allele-exchange method [29]. Second, the Zeo cassette was excised, leaving one FRT site in place of the chrI-4 sequence. To provide homology for the allele exchange, ∼one kb natural flank of chrI-4 followed by a FRT site was cloned upstream of the Zeo cassette in pEM7-Zeo vector that resulted in pBJH242. Likewise, another FRT site followed by ∼one kb of natural sequences at the other flank chrI-4 was cloned downstream of the Zeo cassette of pBJH242 that resulted in pBJH245. The cloned region of pBJH245 (upstream flank of chrI-4-FRT-Zeo cassette-FRT-downstream flank of chrI-4) was amplified by PCR and the linear product was introduced by natural transformation to CVC1121, a *hapR*+ derivative of N16961. The transformants were selected for Zeocin resistance, and the replacement of the chrI-4 sequences by the Zeo cassette was confirmed by PCR amplification of the region and sequencing of the PCR product. The Zeo cassette of the resulting Δ*chrI-4*::FRT-Zeo-FRT strain (CVC2540) was excised by further transformation with an unstable plasmid supplying the Flp recombinase (pBLO1218). The plasmid, when not selected, was lost spontaneously from the final strain Δ*chrI-4*::FRT (CVC2542). The desired deletion/substitution was confirmed by PCR amplifying the mutated region and DNA sequencing. The same strategy was used to make Δ*chrII-10*::FRT-Zeo-FRT strain (CVC2565). The fragment used for natural transformation was a PCR product that had ∼one kb homology to the left flank of chrII-10 (coord. 1026995–1027995)-FRT-Zeo-FRT- ∼one kb homology to the right flank of chrII-10 (coord. 1028034–1029033).

### Marker frequency analysis

Frequencies of four markers, *oriI*, *oriII*, *terI* and *terII*, in exponentially growing cells in L broth were determined by qPCR using a PTC-200 Peltier Thermal Cycler (MJ Research) and a LightCycler 480 SYBR Green I Master (Roche) mix, as described [29].

### *β*-galactosidase assay

Fragments containing putative promoters were cloned into a promoter-less *lacZ* containing plasmid (pMLB1109). The resultant plasmids were used to transform *E. coli* BR8706 carrying p*rctB* (pTVC11). Cells harboring these two plasmids were cultivated in L broth containing 0, 0.002 or 0.2% arabinose at 37°C to exponential phase and *β*-galactosidase activity was measured, as described [58].

### Fluorescence microscopy

To localize *oriI* and *oriII*, P1*parS* as a P1*parS*-Km cassette and pMT*parS* as a pMT*parS*-Sp cassette were integrated at approximately +135 kb region on chrI and +40 kb region on chrII, respectively [58]. Both cassettes were inserted into WT (CVC1121) and Δ*chrI-4* (CVC2542) strains, and the resulting strains (CVC2553 and CVC2554) were transformed with a plasmid expressing mCherry-pMT*parB* and *gfp*-P1*parB* (pRN010). Cells were grown in M9+glycerol medium at 37°C to log phase and observed under a fluorescence microscope, as described [58]. Locations of fluorescent foci were measured using the ImageJ (rsb.info.nih.gov/ij/index.html).

## Supporting Information

Figure S1 Test of origin activity from regions containing the newly identified RctB binding sites. (A) The chrII fragment tested spanned the coordinates 1024390 to 1025604; the sequence shown is of the region considered relevant for the origin activity. It contains several Dam methylation sites (in red), the two iterons belonging to chrII-5 and chrII-6 (in blue and underlined), and a putative DNA box (in green) with 3 mismatches to the consensus TTATCCACA. (B) The chrI fragment tested spanned the coordinates 817948 to 818255, which includes the chrI-4 sequences (underlined). The sequence of the minimal region (70 nt) conferring enhancer activity is italicized. (C) The origin activity of the fragments shown in (A) and (B). The activity was tested by transformation of *E. coli* (BR8706) carrying a source of RctB (pTVC11) with plasmids that carried either the chrII fragment (pchrII = pBJH118) or the chrI fragment (pchrI = pBJH197). The p*oriII* plasmid (pTVC31) was used as a positive control. RctB was supplied in one of three concentrations using arabinose at 0, 0.002 or 0.2%. Selection of transforming plasmids was made on LB agar plates containing appropriate antibiotics (Text S1).
(TIF)

Figure S2 Effect of deletion and extra copies of chrII-10 and chrI-4 sites on *V. cholerae* growth. (A) The growth curves in LB containing appropriate antibiotics are shown for the WT (CVC1121) and Δ*chrII-10* mutant (CVC2565) cells transformed with either an empty vector (pTVC243) or the same vector containing chrII-10 (pTVC350). The results show that the absence or extra copies of the chrII-10 has no growth phenotype. (B) Same as (A) except that the site in question in chrI-4. The Δ*chrI-4* mutant was CVC2542, and the empty vector and pchrI-4 were pACYC177 and pBJH188, respectively. These experiments reveal that the chrI-4 function depends on its copy number as the growth inhibition due to pchrI-4 upon deletion of the chrI-4 sequences from the chromosome (compare WT/pchrI-4 vs. Δ*chrI-4*/pchrI-4). (C) Formation of chrII-less cells is not increased upon deletion of the *chrI-4* site. The loss was measured following the CHUB phenotype [36]. Δ*parAB2* cells were used as positive controls for the CHUB phenotype. 500 cells were counted in each case.
(TIF)

Figure S3 The effect of the chrI-4 site on RctB synthesis. RctB protein levels were determined by Western blots in *E. coli* (A) and in *V. cholerae* (B), and the activity of the natural *rctB* promoter, P*_rctB_*, was determined by *lacZ* fusion (C). (A) The cell extracts were from WT (BR8706, lanes 1–6) or Δ*dnaKJ* (BR4392, lanes 7, 8) cells that carried either the empty vector, pTVC243 (lanes marked vector), or the same vector containing chrI-4, pBJH170 (lanes marked pchrI-4). RctB was supplied from pTVC11 in low and high amounts using 0.002 and 0.2% arabinose, respectively. The Western analysis method was as described [38]. The values of [RctB] were relative to those in vector lanes. The cultures used in lanes 1–6 are representative of experiments in Figure 3A, and in lanes 7 and 8 are representative of the experiment in Figure 4D. (B) *V. cholerae* cells were either WT (CVC1121) or the Δ*chrI-4* mutant (CVC2542), and the plasmids were either the empty vector (pACYC177) or pchrI-4 (pBJH188). Unlike the situation in *E. coli*, a small increase in [RctB] is seen upon deletion of the chrI-4 site (lanes 1,2) and a slight decrease upon providing extra copies of the same site (lanes 3,4). The mean ± standard deviation was calculated from three independent experiments. (C) The P*_rctB_* activity was determined in transformants of *V. cholerae* WT (CVC1121) and the Δ*chrI-4* mutant (CVC2542) using a P*_rctB_-lacZ* fusion-carrying plasmid (pTVC500).
(TIF)

Figure S4 Conservation of the chrI site sequence in the *Vibrio* family. Conservation was apparent in nearly all completely sequenced *Vibrio* genomes recorded in GenBank. The accession numbers of some representative strains are as follows: AE003852.1 for *V. cholerae* (*V. cho*), CP001805.1 for *V. sp.* Ex25 (*V. sp.*), AE016795.3 for *V. vulnificus* (*V. vul*), CP002284.1 for *V. anguillarum* (*V. ang*), CP000789.1 for *V. harveyi* (*V. har*), BA000031.2 for *V. parahaemolyticus* (*V. par*), CP002377.1 for *V. furnissii* (*V. fur*), FM954972.2 for *V. splendidus* (*V. spl*), CP001139.1 for *V. fischeri* (*V. fis*), FM178379.1 for *Aliivibrio salmonicida* (*A. sal*), and CR378665.1 for *Photobacterium profundum* (*P. pro*).
(TIF)

Figure S5 Test of RctB binding to the chrI-4 site in supercoiled form by EMSA. (A) RctB binding to a supercoiled empty vector (pBJH251; labeled vector) or the same vector containing chrI-4 (labeled pchrI-4 = pBJH253) or a 39-mer (p39-mer = pBJH252) was tested in the presence of 0, 2, 20 and 200 nM RctB. (B) Reduction of RctB binding to a 39-mer fragment in the presence of the chrI site. Supercoiled empty vector (pTVC243) or the same vector containing chrI-4 (pBJH170) was added as competitor of RctB binding to the 39-mer carrying fragment (probe). The competitor to probe ratio was varied from 0.04 to 1.28. Percent binding ([intensity of bound probe/intensity of {free+bound} probes]×100) was determined using 10 nM RctB. The error bars are from three repeat measurements of band intensities from the same gel.
(TIF)

Figure S6 Importance of bases protected from DNase I cleavage in RctB binding and enhancer function. (A) To test for RctB binding, the protected bases identified in Figure 3C were mutated in the enhancer fragment chrI-9 and the resulting mutant fragment (chrI-9m) present in plasmid (pBJH227) was used in DNase I footprinting in the presence of 20 nM RctB. Other details are same as in Figure 3C. The DNase digestion patterns with and without RctB were considered identical for chrI-9m, although there could be a small difference around coordinate 40. (B) To test for enhancer function, p*oriII* (pTVC35) copy number was measured in *E. coli* in the presence of either the empty vector (pTVC243) or the same vector carrying either the wild type chrI-9 (resulting in pBJH186) or the mutant chrI-9m (resulting in pBJH227). Other details are same as Figure 2A. (C) The enhancer function was also tested by colony size of *V. cholerae* cells transformed with the plasmids used in (B). The transformants were selected on L agar plates with an antibiotic.
(TIF)

Figure S7 Non-essentiality of DnaK in the chrI enhancer-mediated modulation of RctB binding *in vivo*. The binding was monitored by the promoter repression assay using the chrI-4 enhancer and the empty vector and the same vector with chrI-6 were used as negative controls. These and other details are same as in Figure 4C. Promoter activities were determined in WT (BR4389) and in *dnaK7* mutant (BR4390).
(TIF)

Figure S8 Increase of cell length and *oriII* foci separation in *V. cholerae* Δ*chrI-4* cells. The data of Figure 5C was analyzed for cell length (A) and foci separation (B). The data in (B) indicate that in cells with two origin foci, the separation between the foci has become more heterogeneous for *oriII* compared to *oriI*.
(TIF)

Figure S9 The newly identified RctB binding sites do not affect interaction of RctB with ATP. (A) Effect of ATP and ADP on RctB binding to 39-mers. Binding of purified RctB (100 nM) to the 39-mer (1 nM) was determined by EMSA in the presence of 0, 0.1 or 5 mM ATP, or 5 mM ADP, without or with DnaJ and DnaK. The percent of bound DNA ([intensity of the retarded band/combined intensities of free and retarded bands]×100) is shown below the gel lanes. Note that ATP without the chaperones modestly decreases binding while with chaperones dramatically increases binding. The ADP increases binding modestly whether or not chaperones are present. (B) Same as in (A) without the DnaJ and DnaK lanes but additionally 0.5 nM of either a supercoiled empty vector (pTVC243), or the same vector containing chrII-10 (pTVC350) or chrI-4 (pBJH170) was present. Note that the nucleotide effects remain essentially unaltered whether or not the chrII-10 or the chrI-4 sites were present. (C) Effect of newly identified RctB binding sites on ATP hydrolysis by RctB. ATPase activity of purified RctB (0, 0.5, 5 µM) was compared with 5 nM of pTVC243 (vector), pTVC350 (pchrII-10) or pBJH170 (pchrI-4) by a colorimetric assay. RctB heated at 95°C for 10 min (called inactivated RctB) was used as a negative control, and DnaK alone or a mixture of DnaK and DnaJ was used as positive control. The absorbance values were normalized to the absorbance value seen without RctB. The error bars are from three independent experiments. (D) The effect of newly identified RctB binding sites on p*oriII* (pTVC25) replication was determined by measuring its copy number as in Figure 2A. The copy numbers were determined in the presence of WT RctB and an ATP-insensitive mutant RctB, RctBR269S (present in pBJH260). Note that the mutant increases p*oriII* copy number, nonetheless remains sensitive to the inhibitory effect of chrII-10 and enhancing effect of chrI-4.
(TIF)

Figure S10 The chrI enhancer site does not affect RctB dimerization. (A) The dimerization was assayed in a bacterial two-hybrid system after fusing RctB to T18 and T25 fragments of a bacterial adenylate cyclase (resulting in T18-RctB and T25-RctB fusion proteins). Functional complementation between the fusion proteins was determined by measuring *β*-galactosidase activity (yellow bars). The activities between a T18 or T25fragment and one of the RctB fusion proteins were used as negative controls (white and hatched bars). The activities were determined either in the presence of an empty vector (pGB2) or the same vector containing chrI-4 (pBJH195). The fusion proteins were induced with two different concentrations of IPTG. (B) RctB dimerization was assayed after fusing the protein to the DNA binding domain of λ repressor (λ*cI*_N_) and measuring the repressor activity on the λ*P_R_* promoter (yellow bars). The repression by λ*cI*_N_ alone was used as negative control (white bars). Two different IPTG concentrations were used to induce the synthesis of fusion proteins. The vector and pchrI-4 plasmids are same as in (A). The repression was calculated by normalizing to the λ*P_R_* activities in the absence of IPTG.
(TIF)

Figure S11 Non-essentiality of the promoter activity of the chrI site for the enhancer function. (A) ChrI-4 fragment sequence showing the presence of putative −35 and −10 promoter elements (underlined) within the minimal region (chrI-9) required for the enhancer function. (B) The promoter activities were determined for five fragments (chrI-4, chrI-9, chrI-9m1, chrI-9m2 and chrI-9m1+m2), after fusion to *lacZ* gene (resulting in pBJH232, pBJH223, pBJH228, pBJH229, and pBJH230, respectively) and by measuring *β*-galactosidase activity. chrI-9m1, chrI-9m2 and chrI-9m3 are mutants of chrI-9 where the −35, −10 and both −35 and −10 elements, respectively, are mutated. (C) Lack of correlation of replication enhancer activity with promoter activity of the chrI sites. The enhancer activity was tested by p*oriII* (pTVC35) copy number measurements in the presence of pRctB (pTVC11), whose synthesis was induced with 0.2% arabinose. The fragments were either chrI-4 or chrI-9, or their mutant derivatives carrying mutations m1, m2 and m1+m2, as in (B). Plasmids with m1, m2 and m1+m2 in chrI-4 were pBJH238, pBJH239 and pBJH240, respectively, and with the same mutations in chrI-9 were pBJH247, pBJH248 and pBJH249, respectively. Other details are same as Figure 2A.
(TIF)

Table S1 Effect of putative RctB binding sites from chrII on p*oriII* copy number *in trans*.
(DOCX)

Table S2 Effect of putative RctB binding sites from chrI on p*oriII* copy number *in trans*.
(DOCX)

Table S3 Bacterial strains and plasmids used in this study.
(DOCX)

Text S1 Supporting Materials and Methods.
(DOCX)
