# Supplementary figures and images for: Chromosome I Controls Chromosome II Replication in Vibrio cholerae
Source: PLoS Genet. 2014 Feb 27;10(2):e1004184. doi: 10.1371/journal.pgen.1004184 (PMC3937223; doi:10.1371/journal.pgen.1004184)

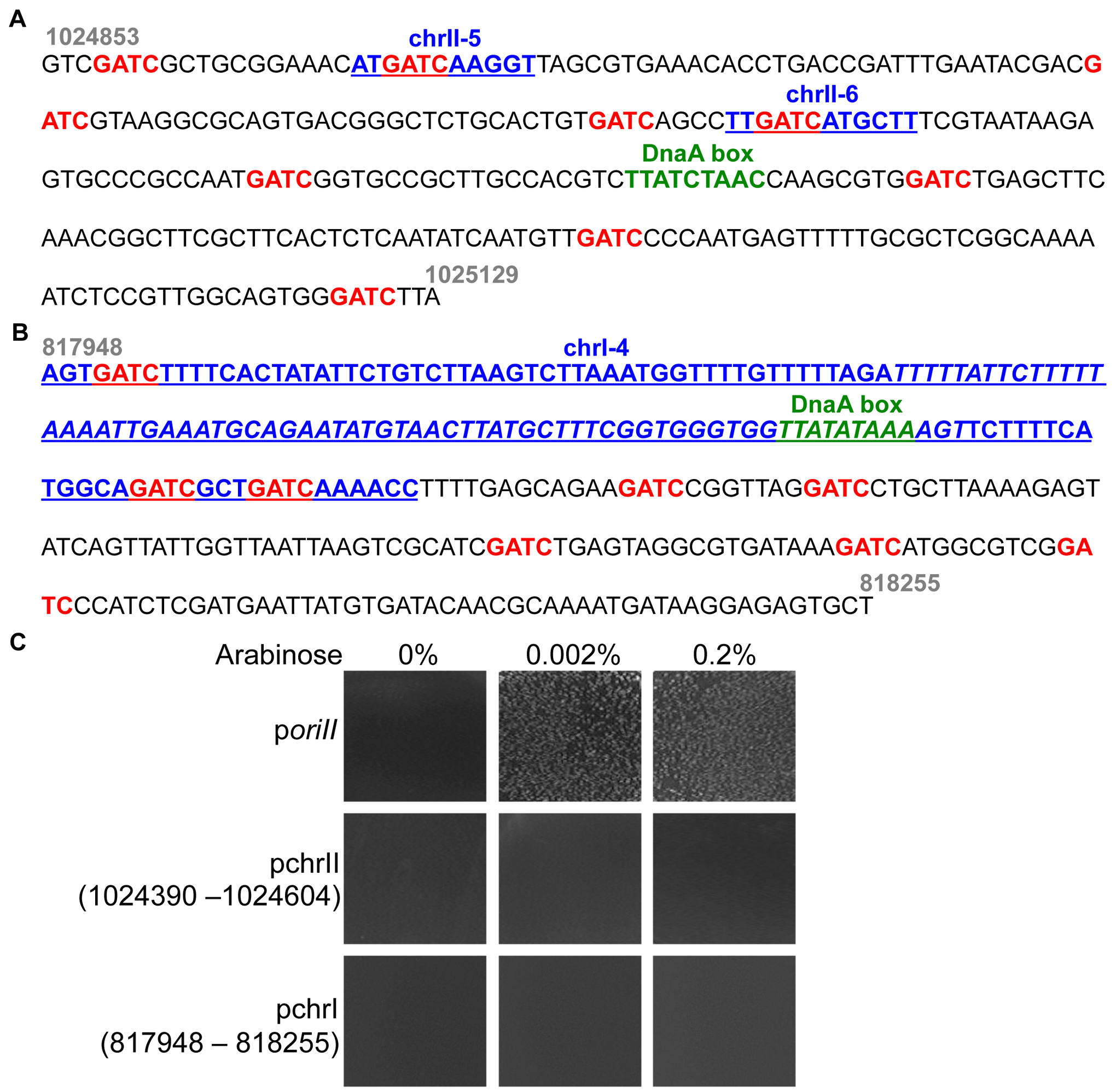

Supplement: Figure S1 — Test of origin activity from regions containing the newly identified RctB binding sites. (A) The chrII fragment tested spanned the coordinates 1024390 to 1025604; the sequence shown is of the region considered relevant for the origin activity. It contains several Dam methylation sites (in red), the two iterons belonging to chrII-5 and chrII-6 (in blue and underlined), and a putative DNA box (in green) with 3 mismatches to the consensus TTATCCACA. (B) The chrI fragment tested spanned the coordinates 817948 to 818255, which includes the chrI-4 sequences (underlined). The sequence of the minimal region (70 nt) conferring enhancer activity is italicized. (C) The origin activity of the fragments shown in (A) and (B). The activity was tested by transformation of E. coli (BR8706) carrying a source of RctB (pTVC11) with plasmids that carried either the chrII fragment (pchrII = pBJH118) or the chrI fragment (pchrI = pBJH197). The poriII plasmid (pTVC31) was used as a positive control. RctB was supplied in one of three concentrations using arabinose at 0, 0.002 or 0.2%. Selection of transforming plasmids was made on LB agar plates containing appropriate antibiotics (Text S1). (TIF) [file pgen.1004184.s001.tif]

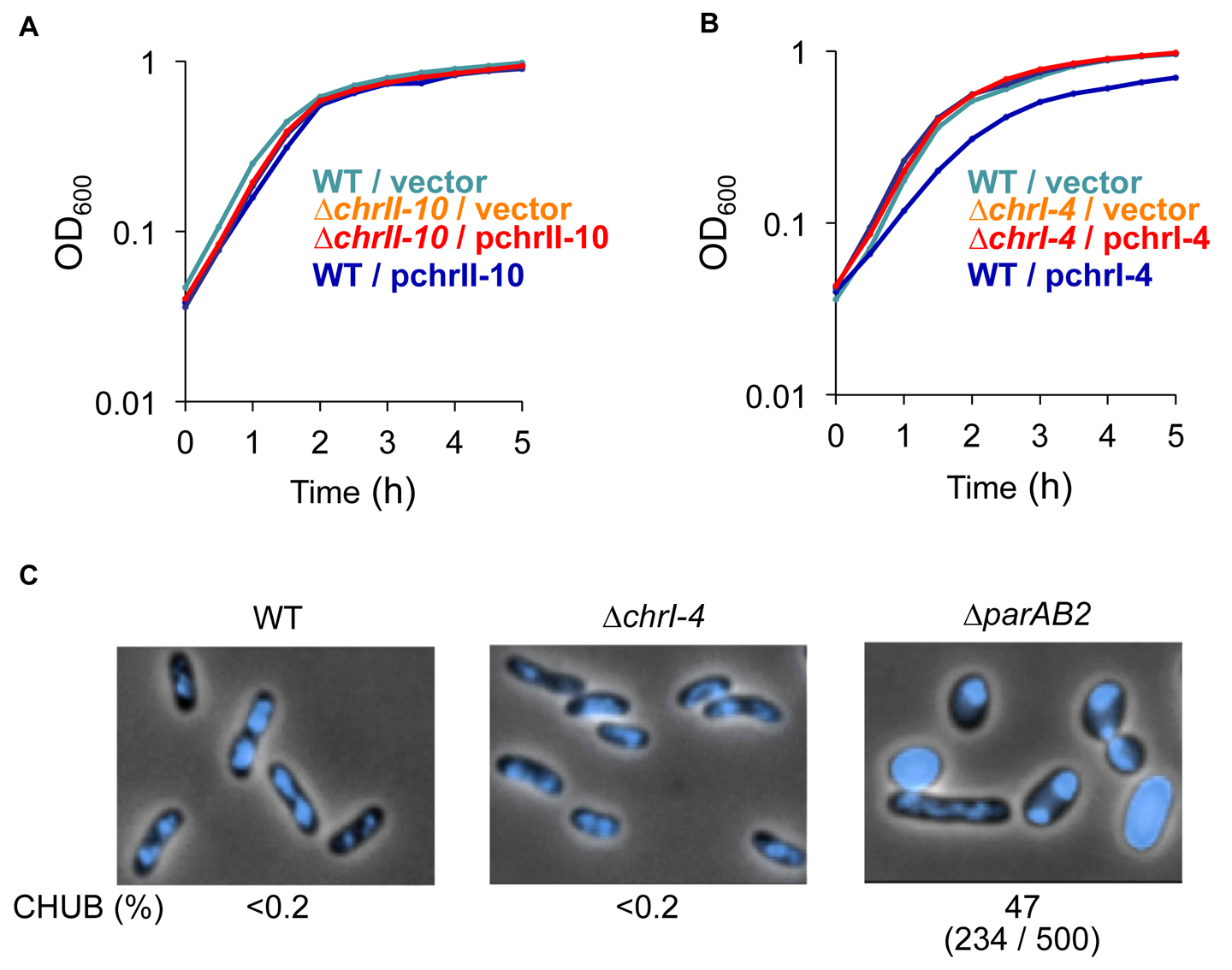

Supplement: Figure S2 — Effect of deletion and extra copies of chrII-10 and chrI-4 sites on V. cholerae growth. (A) The growth curves in LB containing appropriate antibiotics are shown for the WT (CVC1121) and ΔchrII-10 mutant (CVC2565) cells transformed with either an empty vector (pTVC243) or the same vector containing chrII-10 (pTVC350). The results show that the absence or extra copies of the chrII-10 has no growth phenotype. (B) Same as (A) except that the site in question in chrI-4. The ΔchrI-4 mutant was CVC2542, and the empty vector and pchrI-4 were pACYC177 and pBJH188, respectively. These experiments reveal that the chrI-4 function depends on its copy number as the growth inhibition due to pchrI-4 upon deletion of the chrI-4 sequences from the chromosome (compare WT/pchrI-4 vs. ΔchrI-4/pchrI-4). (C) Formation of chrII-less cells is not increased upon deletion of the chrI-4 site. The loss was measured following the CHUB phenotype [36]. ΔparAB2 cells were used as positive controls for the CHUB phenotype. 500 cells were counted in each case. (TIF) [file pgen.1004184.s002.tif]

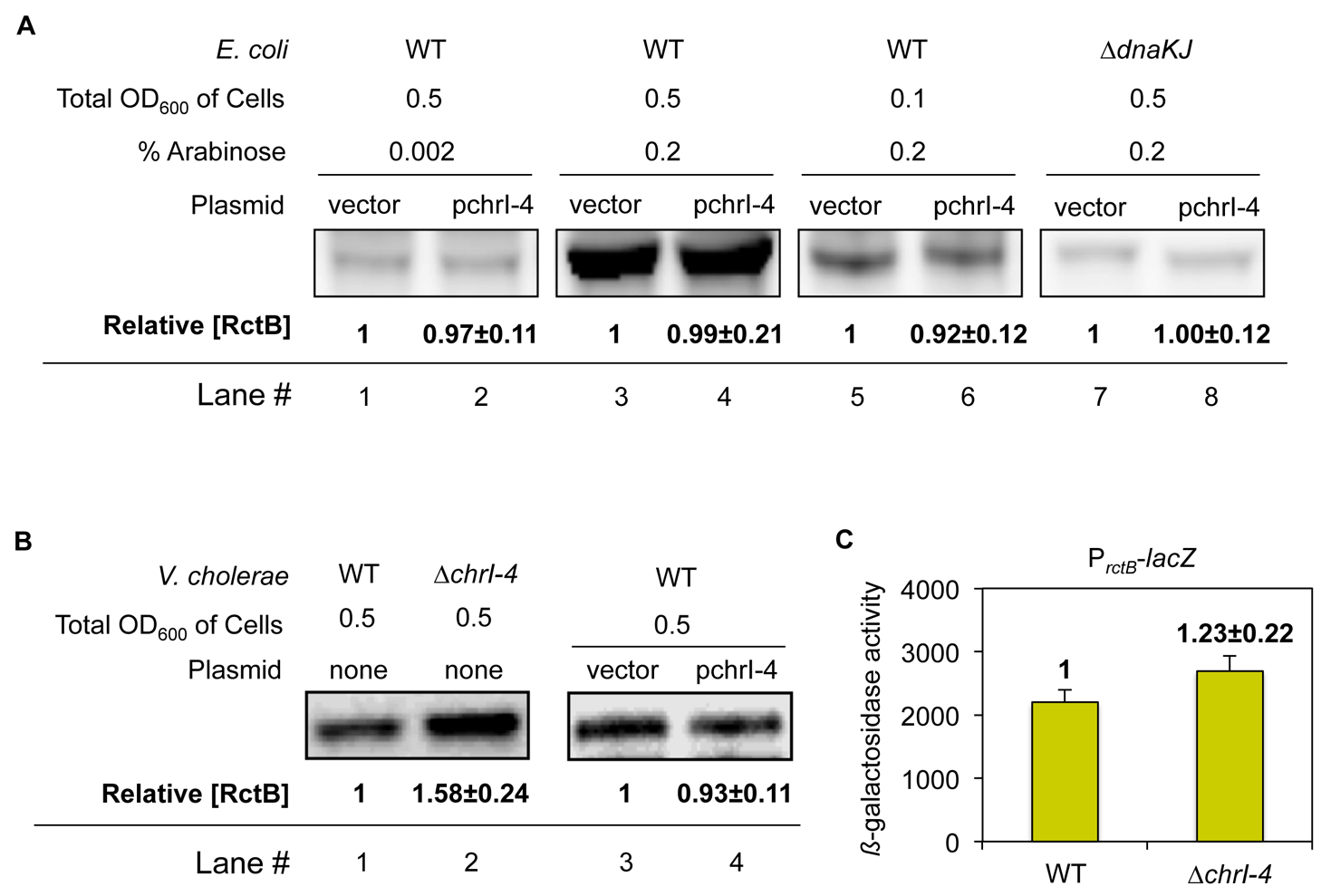

Supplement: Figure S3 — The effect of the chrI-4 site on RctB synthesis. RctB protein levels were determined by Western blots in E. coli (A) and in V. cholerae (B), and the activity of the natural rctB promoter, PrctB, was determined by lacZ fusion (C). (A) The cell extracts were from WT (BR8706, lanes 1–6) or ΔdnaKJ (BR4392, lanes 7, 8) cells that carried either the empty vector, pTVC243 (lanes marked vector), or the same vector containing chrI-4, pBJH170 (lanes marked pchrI-4). RctB was supplied from pTVC11 in low and high amounts using 0.002 and 0.2% arabinose, respectively. The Western analysis method was as described [38]. The values of [RctB] were relative to those in vector lanes. The cultures used in lanes 1–6 are representative of experiments in Figure 3A, and in lanes 7 and 8 are representative of the experiment in Figure 4D. (B) V. cholerae cells were either WT (CVC1121) or the ΔchrI-4 mutant (CVC2542), and the plasmids were either the empty vector (pACYC177) or pchrI-4 (pBJH188). Unlike the situation in E. coli, a small increase in [RctB] is seen upon deletion of the chrI-4 site (lanes 1,2) and a slight decrease upon providing extra copies of the same site (lanes 3,4). The mean ± standard deviation was calculated from three independent experiments. (C) The PrctB activity was determined in transformants of V. cholerae WT (CVC1121) and the ΔchrI-4 mutant (CVC2542) using a PrctB-lacZ fusion-carrying plasmid (pTVC500). (TIF) [file pgen.1004184.s003.tif]

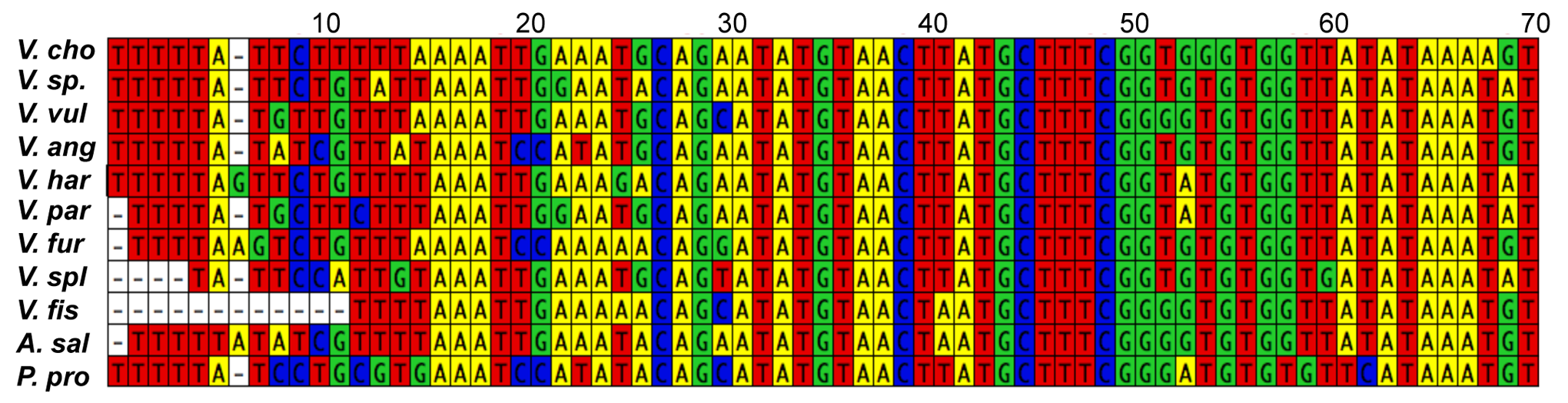

Supplement: Figure S4 — Conservation of the chrI site sequence in the Vibrio family. Conservation was apparent in nearly all completely sequenced Vibrio genomes recorded in GenBank. The accession numbers of some representative strains are as follows: AE003852.1 for V. cholerae (V. cho), CP001805.1 for V. sp. Ex25 (V. sp.), AE016795.3 for V. vulnificus (V. vul), CP002284.1 for V. anguillarum (V. ang), CP000789.1 for V. harveyi (V. har), BA000031.2 for V. parahaemolyticus (V. par), CP002377.1 for V. furnissii (V. fur), FM954972.2 for V. splendidus (V. spl), CP001139.1 for V. fischeri (V. fis), FM178379.1 for Aliivibrio salmonicida (A. sal), and CR378665.1 for Photobacterium profundum (P. pro). (TIF) [file pgen.1004184.s004.tif]

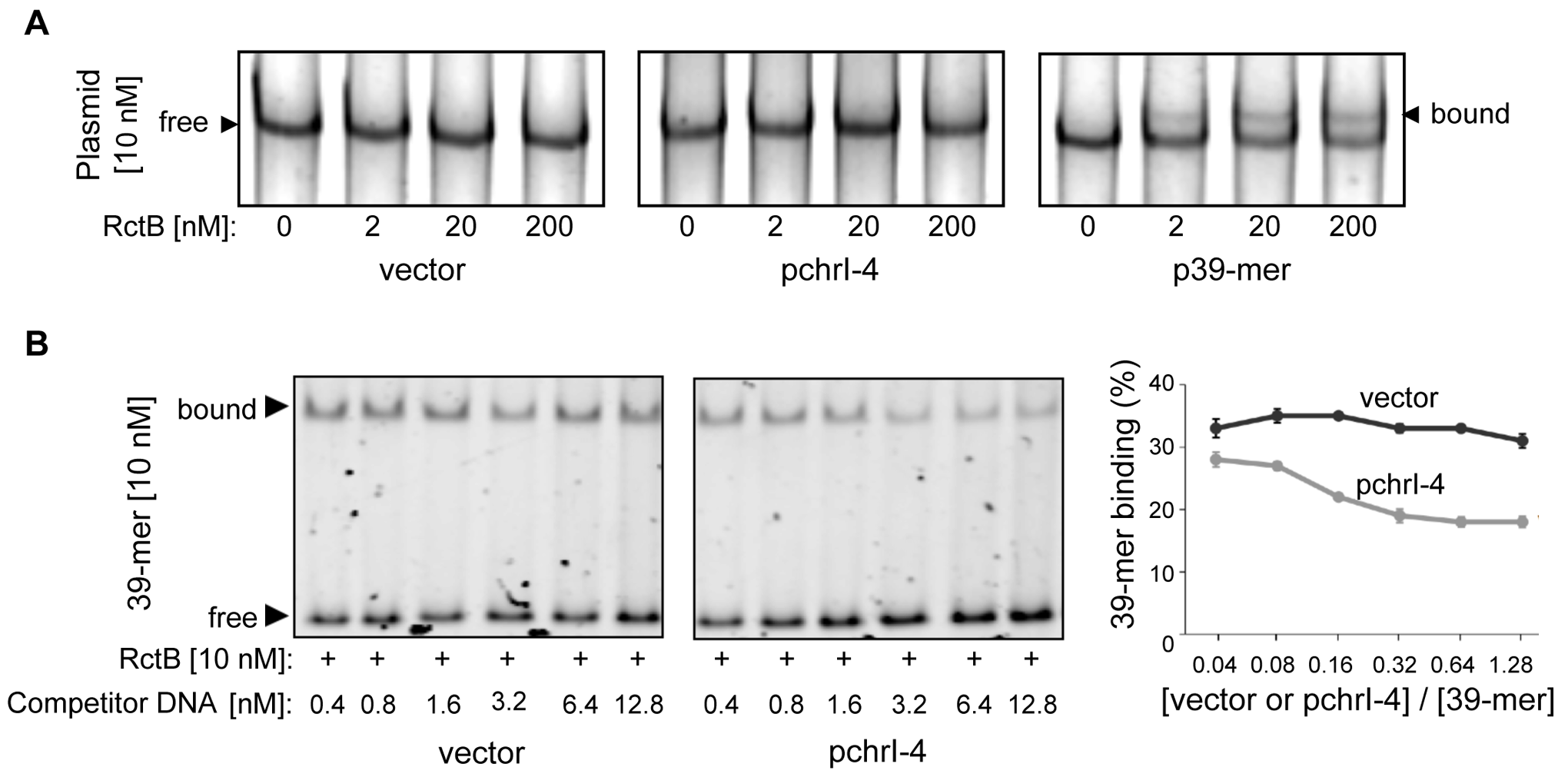

Supplement: Figure S5 — Test of RctB binding to the chrI-4 site in supercoiled form by EMSA. (A) RctB binding to a supercoiled empty vector (pBJH251; labeled vector) or the same vector containing chrI-4 (labeled pchrI-4 = pBJH253) or a 39-mer (p39-mer = pBJH252) was tested in the presence of 0, 2, 20 and 200 nM RctB. (B) Reduction of RctB binding to a 39-mer fragment in the presence of the chrI site. Supercoiled empty vector (pTVC243) or the same vector containing chrI-4 (pBJH170) was added as competitor of RctB binding to the 39-mer carrying fragment (probe). The competitor to probe ratio was varied from 0.04 to 1.28. Percent binding ([intensity of bound probe/intensity of {free+bound} probes]×100) was determined using 10 nM RctB. The error bars are from three repeat measurements of band intensities from the same gel. (TIF) [file pgen.1004184.s005.tif]

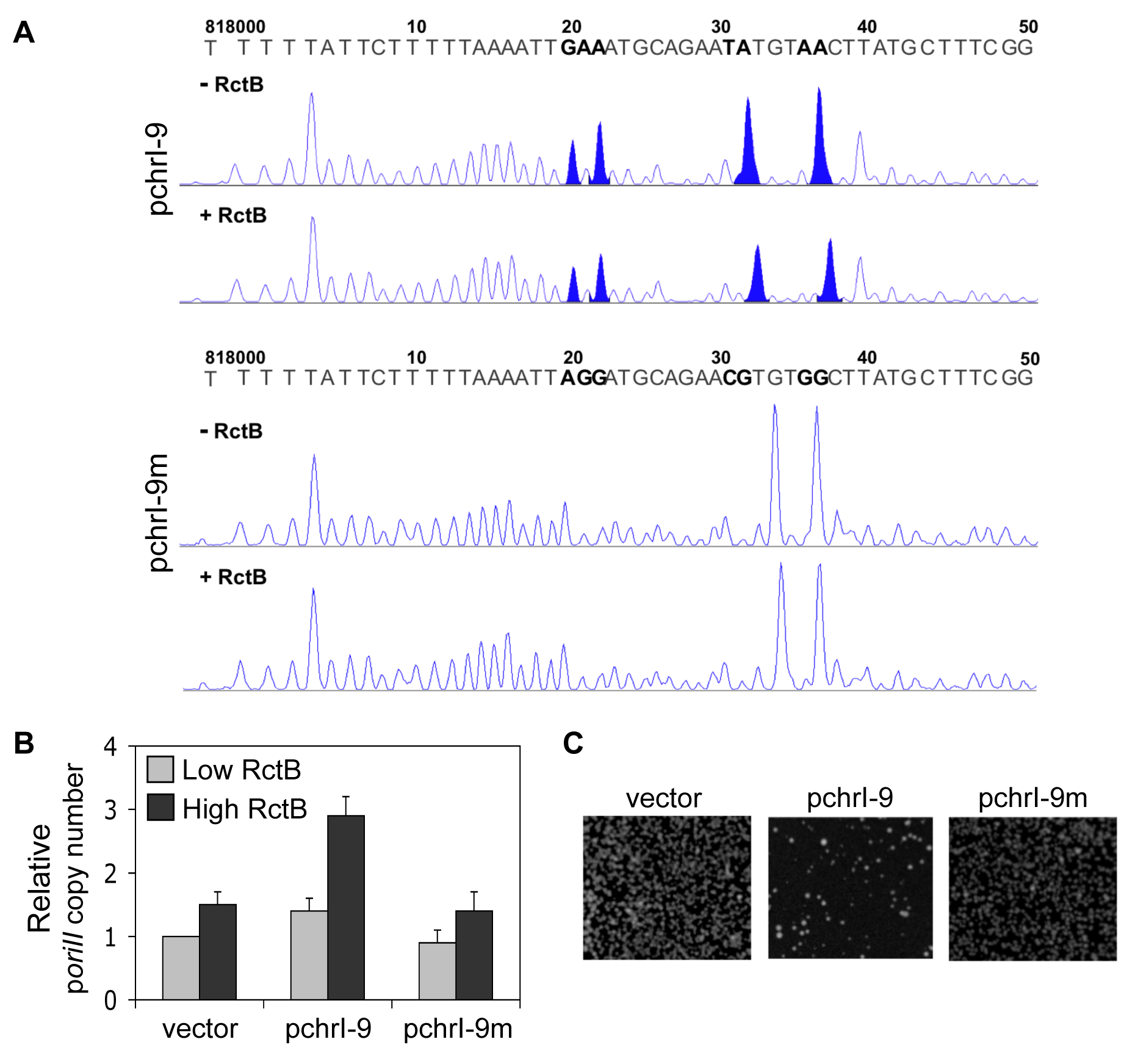

Supplement: Figure S6 — Importance of bases protected from DNase I cleavage in RctB binding and enhancer function. (A) To test for RctB binding, the protected bases identified in Figure 3C were mutated in the enhancer fragment chrI-9 and the resulting mutant fragment (chrI-9m) present in plasmid (pBJH227) was used in DNase I footprinting in the presence of 20 nM RctB. Other details are same as in Figure 3C. The DNase digestion patterns with and without RctB were considered identical for chrI-9m, although there could be a small difference around coordinate 40. (B) To test for enhancer function, poriII (pTVC35) copy number was measured in E. coli in the presence of either the empty vector (pTVC243) or the same vector carrying either the wild type chrI-9 (resulting in pBJH186) or the mutant chrI-9m (resulting in pBJH227). Other details are same as Figure 2A. (C) The enhancer function was also tested by colony size of V. cholerae cells transformed with the plasmids used in (B). The transformants were selected on L agar plates with an antibiotic. (TIF) [file pgen.1004184.s006.tif]

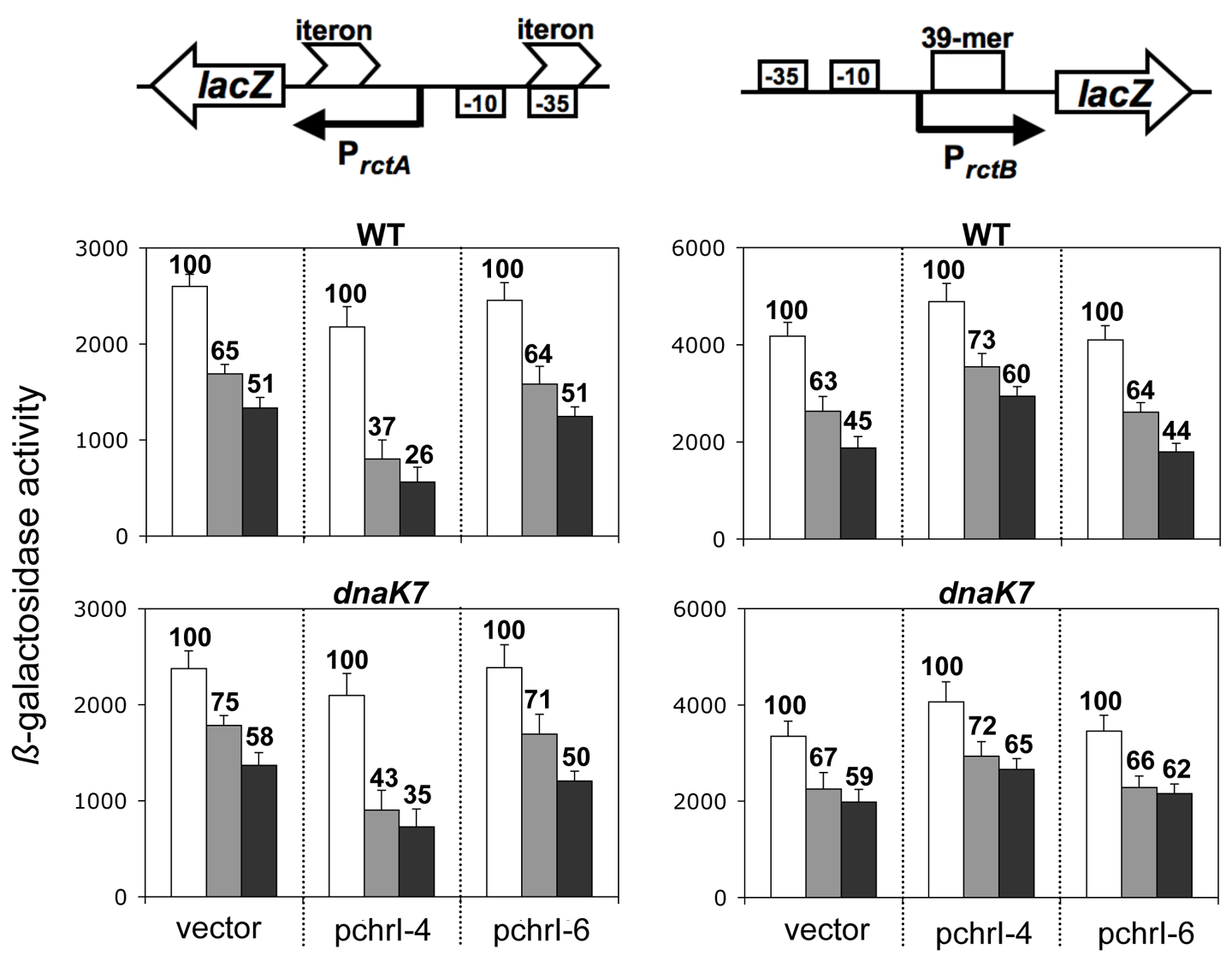

Supplement: Figure S7 — Non-essentiality of DnaK in the chrI enhancer-mediated modulation of RctB binding in vivo. The binding was monitored by the promoter repression assay using the chrI-4 enhancer and the empty vector and the same vector with chrI-6 were used as negative controls. These and other details are same as in Figure 4C. Promoter activities were determined in WT (BR4389) and in dnaK7 mutant (BR4390). (TIF) [file pgen.1004184.s007.tif]

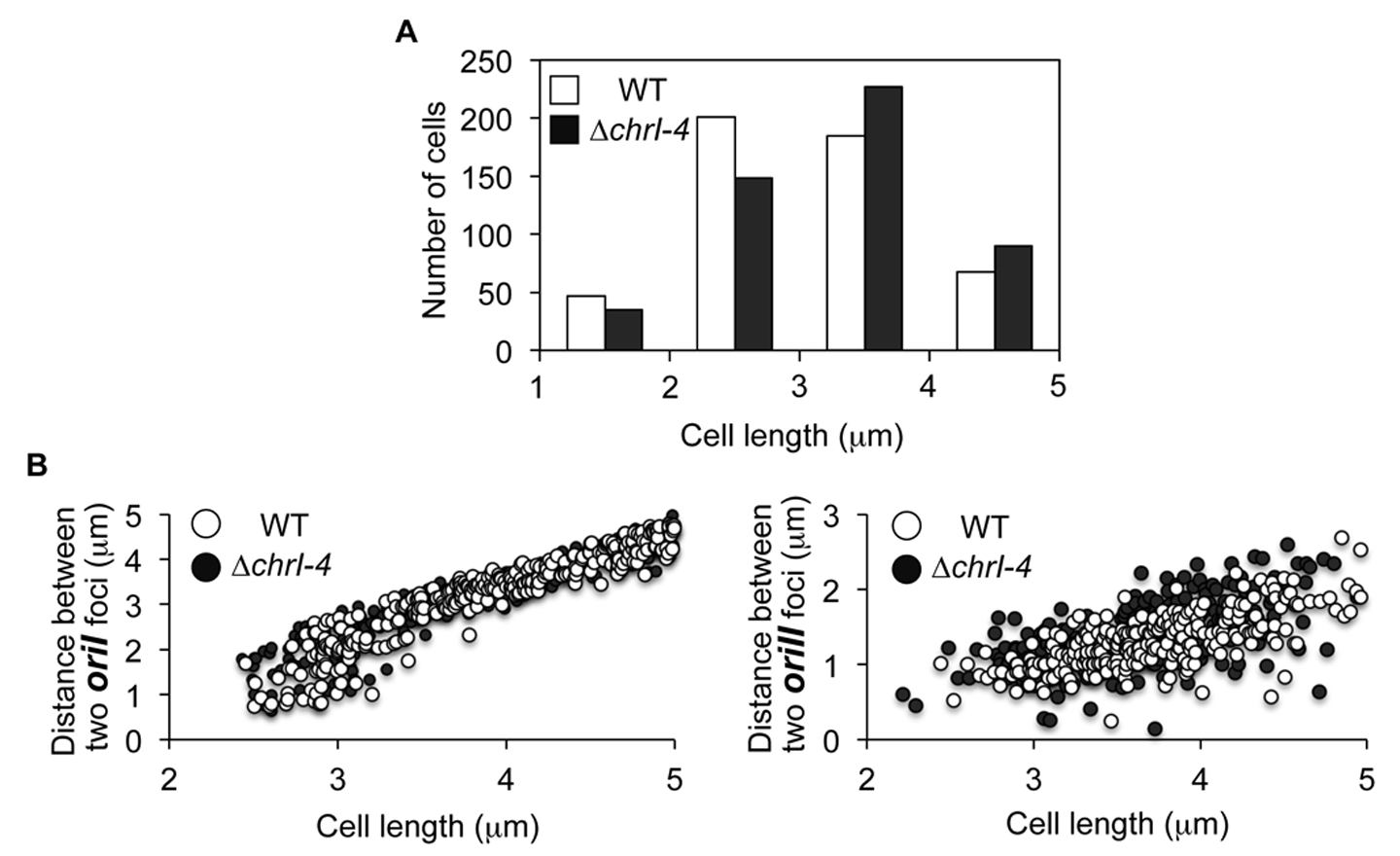

Supplement: Figure S8 — Increase of cell length and oriII foci separation in V. cholerae ΔchrI-4 cells. The data of Figure 5C was analyzed for cell length (A) and foci separation (B). The data in (B) indicate that in cells with two origin foci, the separation between the foci has become more heterogeneous for oriII compared to oriI. (TIF) [file pgen.1004184.s008.tif]

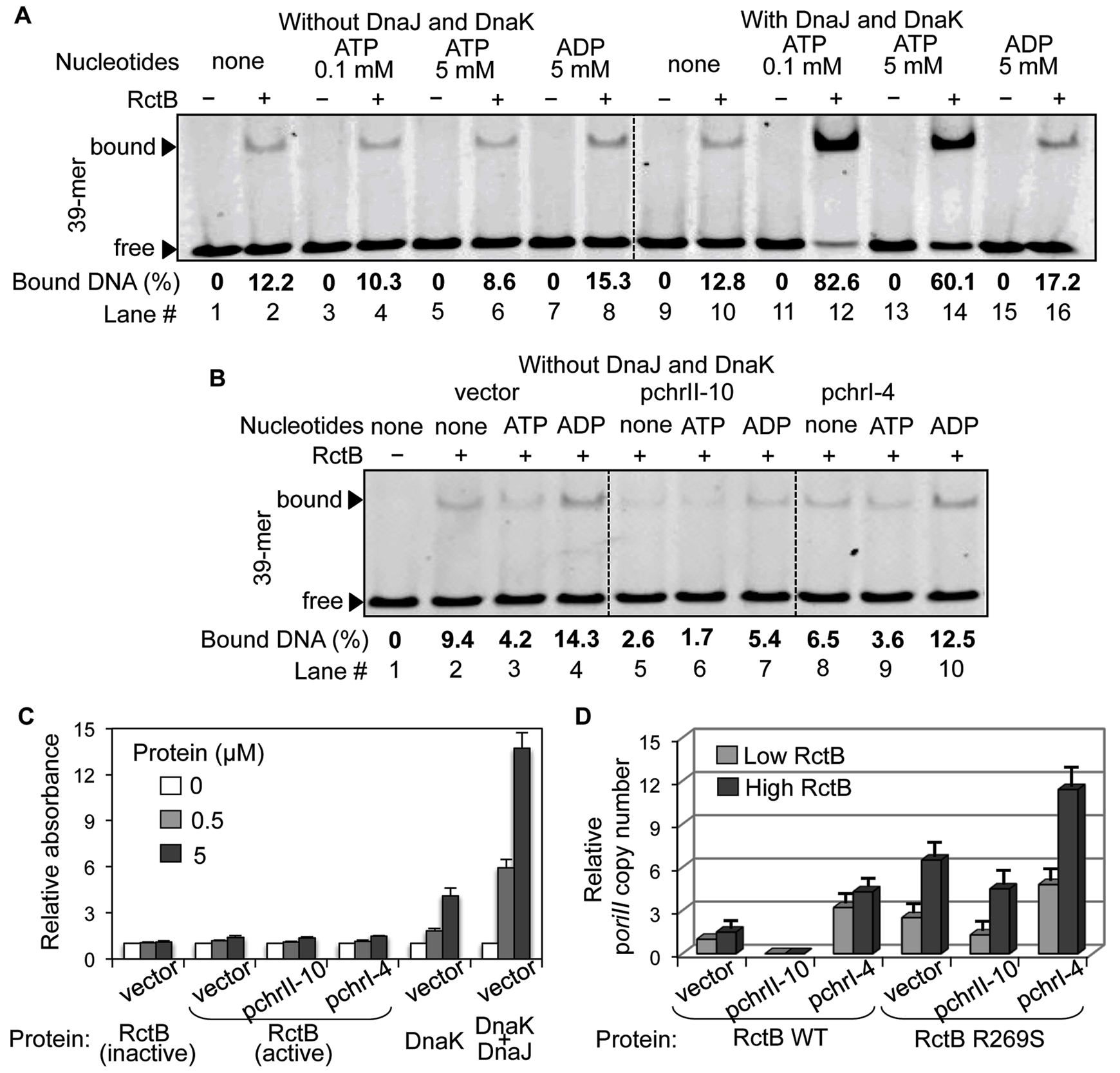

Supplement: Figure S9 — The newly identified RctB binding sites do not affect interaction of RctB with ATP. (A) Effect of ATP and ADP on RctB binding to 39-mers. Binding of purified RctB (100 nM) to the 39-mer (1 nM) was determined by EMSA in the presence of 0, 0.1 or 5 mM ATP, or 5 mM ADP, without or with DnaJ and DnaK. The percent of bound DNA ([intensity of the retarded band/combined intensities of free and retarded bands]×100) is shown below the gel lanes. Note that ATP without the chaperones modestly decreases binding while with chaperones dramatically increases binding. The ADP increases binding modestly whether or not chaperones are present. (B) Same as in (A) without the DnaJ and DnaK lanes but additionally 0.5 nM of either a supercoiled empty vector (pTVC243), or the same vector containing chrII-10 (pTVC350) or chrI-4 (pBJH170) was present. Note that the nucleotide effects remain essentially unaltered whether or not the chrII-10 or the chrI-4 sites were present. (C) Effect of newly identified RctB binding sites on ATP hydrolysis by RctB. ATPase activity of purified RctB (0, 0.5, 5 µM) was compared with 5 nM of pTVC243 (vector), pTVC350 (pchrII-10) or pBJH170 (pchrI-4) by a colorimetric assay. RctB heated at 95°C for 10 min (called inactivated RctB) was used as a negative control, and DnaK alone or a mixture of DnaK and DnaJ was used as positive control. The absorbance values were normalized to the absorbance value seen without RctB. The error bars are from three independent experiments. (D) The effect of newly identified RctB binding sites on poriII (pTVC25) replication was determined by measuring its copy number as in Figure 2A. The copy numbers were determined in the presence of WT RctB and an ATP-insensitive mutant RctB, RctBR269S (present in pBJH260). Note that the mutant increases poriII copy number, nonetheless remains sensitive to the inhibitory effect of chrII-10 and enhancing effect of chrI-4. (TIF) [file pgen.1004184.s009.tif]

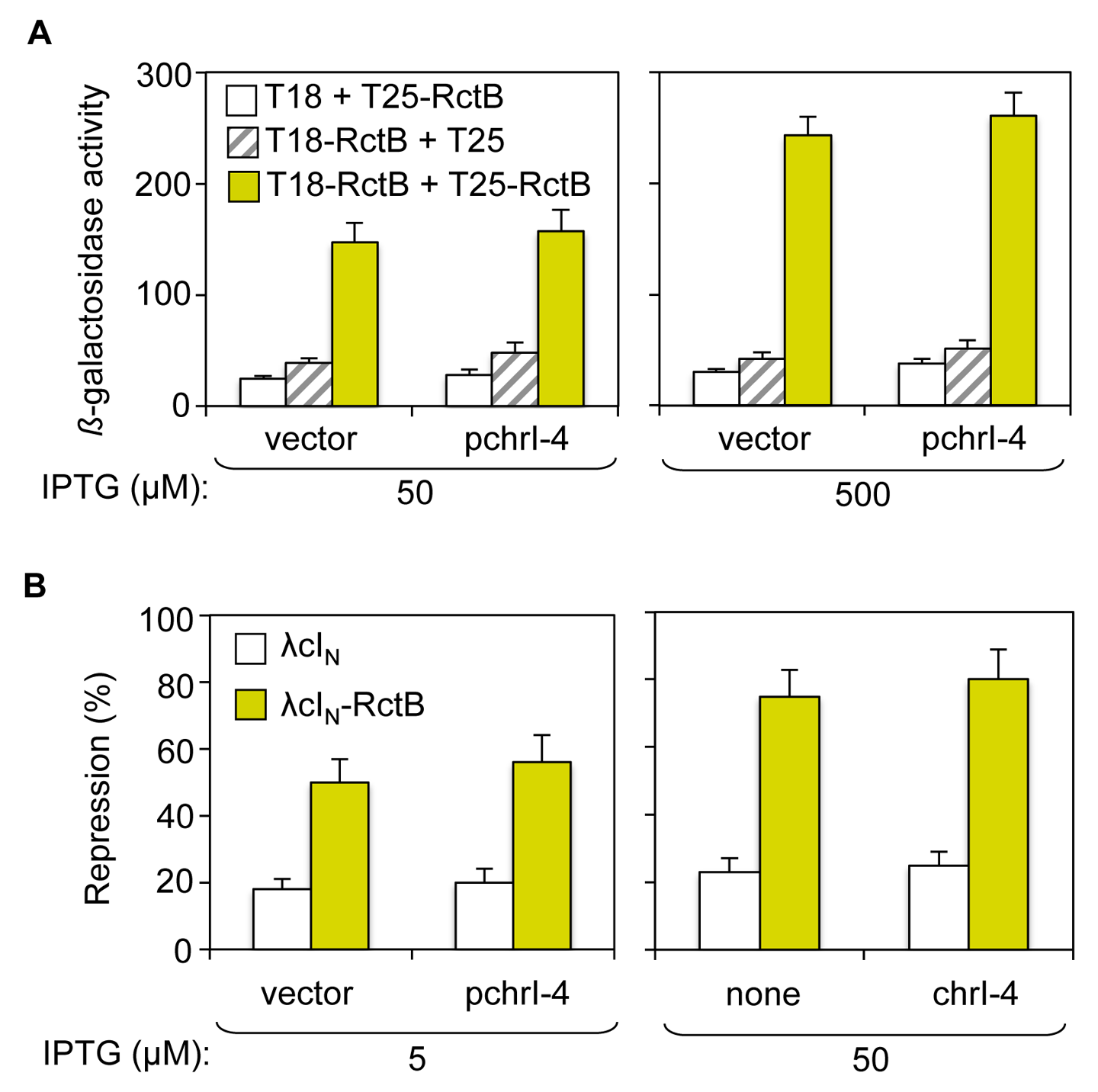

Supplement: Figure S10 — The chrI enhancer site does not affect RctB dimerization. (A) The dimerization was assayed in a bacterial two-hybrid system after fusing RctB to T18 and T25 fragments of a bacterial adenylate cyclase (resulting in T18-RctB and T25-RctB fusion proteins). Functional complementation between the fusion proteins was determined by measuring β-galactosidase activity (yellow bars). The activities between a T18 or T25fragment and one of the RctB fusion proteins were used as negative controls (white and hatched bars). The activities were determined either in the presence of an empty vector (pGB2) or the same vector containing chrI-4 (pBJH195). The fusion proteins were induced with two different concentrations of IPTG. (B) RctB dimerization was assayed after fusing the protein to the DNA binding domain of λ repressor (λcI N) and measuring the repressor activity on the λPR promoter (yellow bars). The repression by λcI N alone was used as negative control (white bars). Two different IPTG concentrations were used to induce the synthesis of fusion proteins. The vector and pchrI-4 plasmids are same as in (A). The repression was calculated by normalizing to the λPR activities in the absence of IPTG. (TIF) [file pgen.1004184.s010.tif]

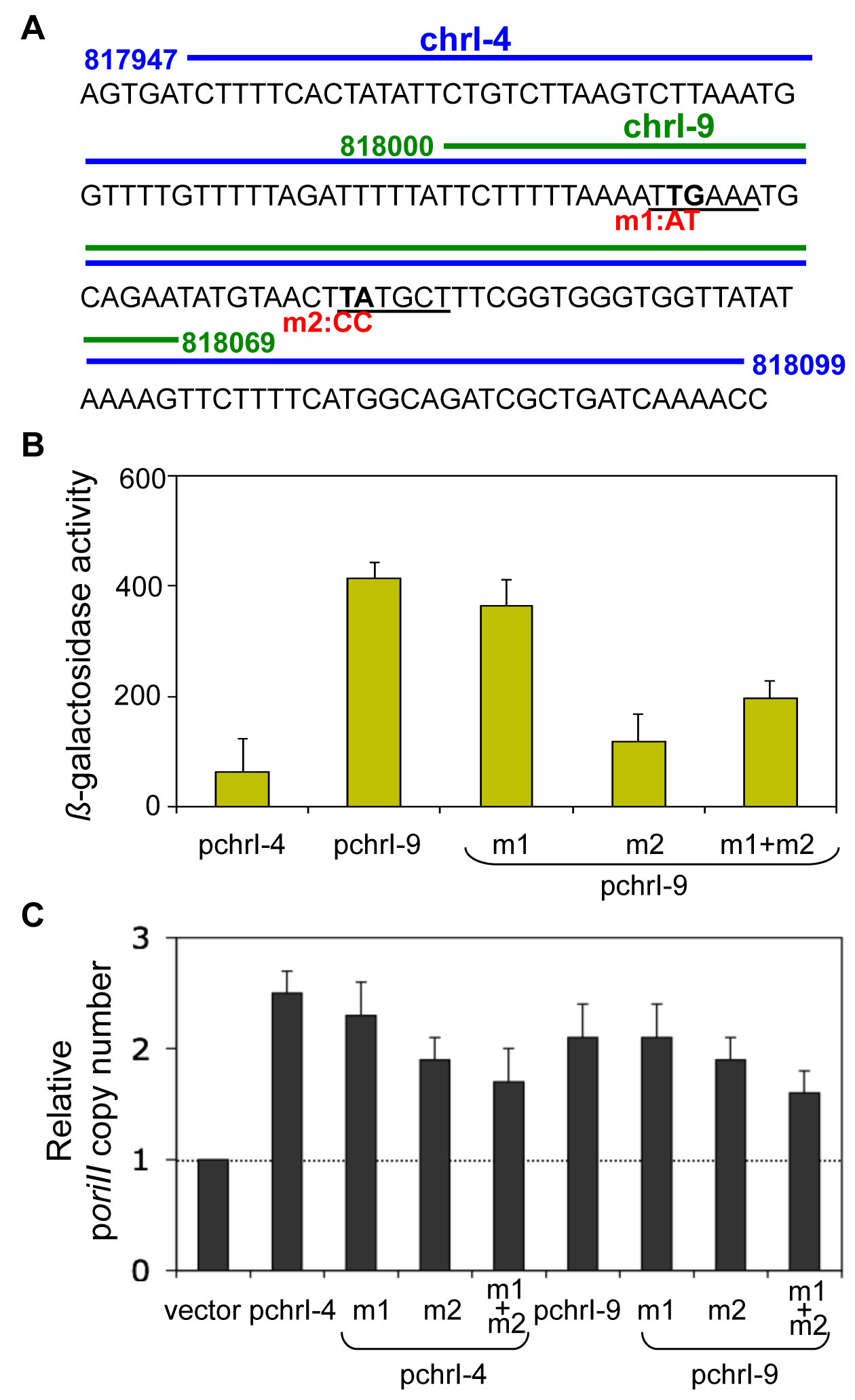

Supplement: Figure S11 — Non-essentiality of the promoter activity of the chrI site for the enhancer function. (A) ChrI-4 fragment sequence showing the presence of putative −35 and −10 promoter elements (underlined) within the minimal region (chrI-9) required for the enhancer function. (B) The promoter activities were determined for five fragments (chrI-4, chrI-9, chrI-9m1, chrI-9m2 and chrI-9m1+m2), after fusion to lacZ gene (resulting in pBJH232, pBJH223, pBJH228, pBJH229, and pBJH230, respectively) and by measuring β-galactosidase activity. chrI-9m1, chrI-9m2 and chrI-9m3 are mutants of chrI-9 where the −35, −10 and both −35 and −10 elements, respectively, are mutated. (C) Lack of correlation of replication enhancer activity with promoter activity of the chrI sites. The enhancer activity was tested by poriII (pTVC35) copy number measurements in the presence of pRctB (pTVC11), whose synthesis was induced with 0.2% arabinose. The fragments were either chrI-4 or chrI-9, or their mutant derivatives carrying mutations m1, m2 and m1+m2, as in (B). Plasmids with m1, m2 and m1+m2 in chrI-4 were pBJH238, pBJH239 and pBJH240, respectively, and with the same mutations in chrI-9 were pBJH247, pBJH248 and pBJH249, respectively. Other details are same as Figure 2A. (TIF) [file pgen.1004184.s011.tif]
