# Supplementary material for: Chromosome I Controls Chromosome II Replication in Vibrio cholerae
Source: PLoS Genet. 2014 Feb 27;10(2):e1004184. doi: 10.1371/journal.pgen.1004184 (PMC3937223; doi:10.1371/journal.pgen.1004184)
Supplement: Table S1 — Effect of putative RctB binding sites from chrII on poriII copy number in trans. (DOCX) [file pgen.1004184.s012.docx]

**Table S1.** Effect of putative RctB binding sites from chrII on p*oriII* copy number *in trans.*

| **Site name (type)^a^** | **Site coordinates**  **(chrII)** | **Site sequence in the region homologous to**  **an iteron or a 39-mer^b^** | **Relative copy number of p*oriII*^c^** | |
| --- | --- | --- | --- | --- |
|  |  |  | **Low RctB** | **High RctB** |
| None |  |  | 1 | 1.5 ± 0.2 |
| Iteron | 572 - 595 | A**TGATCA**TGCTT | < 0.1 | 0.5 ± 0.2 |
| 39-mer | 448 - 487 | **CGGAAGCATG**TAAATTCAT  TATCAATTTA**CGGTCGATG** | < 0.1 | < 0.1 |
| chrII-1 (iteron) | 956828 - 956851 | A**TGATCA**TGCTT | < 0.1 | 0.6 ± 0.2 |
| chrII-2 (39-mer) | 1023582 - 1023630 | **CGG**TAAAGATAGTGAGGCC  CGCCCGCTTT**CGG**C**C**CCGT | 1.1 ± 0.1 | 1.6 ± 0.2 |
| chrII-3 (39-mer) | 1024309 - 1024391 | **CGGA**T**G**AGAAGTGAGTGCG  CTGCCGATAT**CGG**CACCGC | 1.0 ± 0.1 | 1.5 ± 0.2 |
| chrII-4 (iteron) | 1024600 - 1024622 | T**TGATC**CATCAA | 0.8 ± 0.2 | 1.2 ± 0.2 |
| chrII-5 (iteron) | 1024865 - 1024888 | A**TGATCA**AGGT | < 0.1 | 0.4 ± 0.2 |
| chrII-6 (iteron) | 1024942 - 1024973 | T**TGATCA**TGCTT | < 0.1 | 0.3 ± 0.2 |
| chrII-7 (iteron) | 1024983 - 1025006 | A**TGATC**GGTGCC | 0.9 ± 0.2 | 1.3 ± 0.2 |
| chrII-8 (iteron) | 1027955 - 1027978 | AA**GATC**TTGGCT | 1.1 ± 0.1 | 1.8 ± 0.2 |
| chrII-9 (iteron) | 1027995 - 1028019 | A**TGATCA**AGTG | < 0.1 | 0.4 ± 0.2 |
| chrII-10  (iteron+39-mer) | 1027996 - 1028033 | **CGGAA**CA**ATG**ATCAAGTGC  ACATAAATTA**CGGTC**A**ATG** | < 0.1 | < 0.1 |
| chrII-11 (iteron) | 1030750 - 1030773 | T**TGATCA**TGCTT | < 0.1 | 0.4 ± 0.2 |
| chrII-12 (39-mer) | 1030808 - 1030894 | **CG**TC**A**CTCAAACCAAATACAAAACTGATG**CGG**AACTCA | 1.0 ± 0.1 | 1.7 ± 0.2 |

^a^ The sites were typed by their similarity to an iteron (line 2) or a 39-mer sequences of the origin

(line 3) [1]. Only the hexamer sequence shown in bold is fully conserved among the 11 origin iterons. The 39-mer shown is the strongest replication inhibitor of its kind. Its direct repeats shown in bold are the most conserved sequences among the 39-mers.

^b^The site sequences that matched the consensus iteron hexamer or the direct repeat of the 39-mer sequence are shown in bold.

^c^RctB was supplied at low and high concentrations using arabinose at 0.002% or 0.2%, respectively. The copy numbers of p*oriII* was normalized with respect to the value at low RctB when there were no cloned sites *in trans* (line 1). The standard deviations are from three independent experiments.

**Reference**

1. Venkova-Canova T, Baek JH, Fitzgerald PC, Blokesch M, Chattoraj DK (2013) Evidence for Two Different Regulatory Mechanisms Linking Replication and Segregation of *Vibrio cholerae* Chromosome II. PLoS Genet 9: e1003579.
