# Supplementary material for: Chromosome I Controls Chromosome II Replication in Vibrio cholerae
Source: PLoS Genet. 2014 Feb 27;10(2):e1004184. doi: 10.1371/journal.pgen.1004184 (PMC3937223; doi:10.1371/journal.pgen.1004184)
Supplement: Table S2 — Effect of putative RctB binding sites from chrI on poriII copy number in trans. (DOCX) [file pgen.1004184.s013.docx]

**Table S2.** Effect of putative RctB binding sites from chrI on p*oriII* copy number *in trans.*

| **Site name^a^** | **Site coordinates**  **present** | **Relative copy number of p*oriII*^b^** | |
| --- | --- | --- | --- |
|  |  | **Low RctB** | **High RctB** |
| None |  | 1 | 1.5 ± 0.2 |
| chrI-1 | 818082 - 818105 | 0.8 ± 0.2 | 1.2 ± 0.2 |
| chrI-2 | 817200 - 818899 | 1.8 ± 0.3 | 3.7 ± 0.3 |
| chrI-3 | 817800 - 818099 | 1.5 ± 0.2 | 3.5 ± 0.3 |
| chrI-4 | 817947 - 818099 | 1.7 ± 0.3 | 3.3 ± 0.3 |
| chrI-5 | 818000 - 818099 | 1.5 ± 0.2 | 3.3 ± 0.3 |
| chrI-6 | 818010 - 818099 | 1.2 ± 0.2 | 1.7 ± 0.2 |
| chrI-7 | 818000 - 818086 | 1.5 ± 0.2 | 2.8 ± 0.2 |
| chrI-8 | 818000 - 818079 | 1.5 ± 0.2 | 2.9 ± 0.3 |
| chrI-9 | 818000 - 818069 | 1.4 ± 0.2 | 2.6 ± 0.3 |
| chrI-9m | 818000 – 818069 (mutated) | 0.9 ± 0.2 | 1.4 ± 0.3 |
| chrI-10 | 818000 - 818059 | 1.3 ± 0.3 | 1.9 ± 0.3 |

^a^ Only chrI-1 putative site contained a sequence C**TGATCA**AAAC similar to an iteron sequence. No 39-mer type sequence could be recognized in any of the chrI sites tested.

^b^Copy numbers were measured as described in Table S1.
