# Supplementary material for: Chromosome I Controls Chromosome II Replication in Vibrio cholerae
Source: PLoS Genet. 2014 Feb 27;10(2):e1004184. doi: 10.1371/journal.pgen.1004184 (PMC3937223; doi:10.1371/journal.pgen.1004184)
Supplement: Table S3 — Bacterial strains and plasmids used in this study. (DOCX) [file pgen.1004184.s014.docx]

**Table S3.** Bacterial strains and plasmids used in this study.

| **Strains** | **Relevant characteristics** | **Ref. or Source** | |
| --- | --- | --- | --- |
| BR4392 | Δ(*dnaK-J*)306::miniKan Δ(*srlR-recA*)306::Tn*10*; Km^R^, Tc^R^ | | [[1](#_ENREF_1)] |
| BR4389 | RB85(=*thr leu thi lacY rpsL supE* λ^-^) Δ(*srlR-recA*)306::Tn*10*; Tc^R^ | | [[1](#_ENREF_1)] |
| BR4390 | RB851(=*thr leu thi lacY rpsL supE* *dnaK7* λ^-^) Δ(*srlR-recA*)306::Tn*10*; Tc^R^ | | [[1](#_ENREF_1)] |
| BR6610 | =MC1061(λ202=λP_R_ O_R_2^-^*lacZ*)/F’ *lacI^q^* *lacZ*::Tn*5* | | [[3](#_ENREF_3)] |
| BR8706 | =Stbl2= Δ(*lac-proAB*) Δ(*araFGH*) Δ*araEp* P_CP18_-*araE*; *araE* under constitutive CP18 promoter | | [2] |
| CVC1837 | =BTH101=F^–^ *cya-99 araD139 galE15 galK16 rpsL1 hsdR2 mcrA1 mcrB1*; Str^R^ | | Euromedex |
| CVC209 | *V. cholerae* El Tor N16961; Str^R^ | | M. Waldor |
| CVC1121 | N16961 *hapR^+^ Δdns* | | M. Blokesch |
| CVC2099 | =MCH1= El Tor N16961 with fused chrI & chrII | | [[4](#_ENREF_4)] |
| CVC2540 | CVC1121 Δ*chrI-4*::FRT-*zeo*-FRT; Zeo^R^ | | This work |
| CVC2542 | CVC1121 Δ*chrI-4*::FRT | | This work |
| CVC2553 | CVC1121+P1*parS*-Km (at +135 kb on chrI)+pMT*parS*-Sp (at +40 kb on chrII); Km^R^, Sp^R^ | | This work |
| CVC2554 | CVC2542+P1*parS*-Km (at +135 kb on chrI)+pMT*parS*-Sp (at +40 kb on chrII); Km^R^, Sp^R^ | | This work |
| CVC2558 | =YBB324=N16961 *ΔparAB2*; Sp^R^ | | [[5](#_ENREF_5)] |
| CVC2565 | CVC1121 Δ*chrII-10*::FRT-*zeo*-FRT; Zeo^R^ | | This work |
|  |  | |  |
| **Plasmids** |  | |  |
| pACYC177 | p15A*ori*; Ap^R^, Km^R^ | | NEB |
| pBJH93 | pKT25+*rctB* (coord. 1118–3115); Km^R^ | | [[6](#_ENREF_6)] |
| pBJH95 | pUT18C+*rctB* (coord. 1118–3115); Ap^R^ | | [[6](#_ENREF_6)] |
| pBJH97 | pTVC243+chrII-2 (coord. 1023582–1023630); Cm^R^ | | This work |
| pBJH98 | pTVC243+chrII-3 (coord. 1024309–1024391); Cm^R^ | | This work |
| pBJH100 | pTVC243+chrII-8 (coord. 1027955–1027978); Cm^R^ | | This work |
| pBJH101 | pTVC243+chrII-11 (coord. 1030750–1030773); Cm^R^ | | This work |
| pBJH102 | pTVC243+chrII-12 (coord. 1030808–1030894); Cm^R^ | | This work |
| pBJH118 | pTVC31 Δ*oriII* :: ChrII coord. 1024390–1025604; Ap^R^ | | This work |
| pBJH121 | pTVC243+chrII-5 (coord. 1024865–1024888); Cm^R^ | | This work |
| pBJH122 | pTVC243+chrII-6 (coord. 1024942–1024973); Cm^R^ | | This work |
| pBJH123 | pTVC243+chrII-7 (coord. 1024983–1025006); Cm^R^ | | This work |
| pBJH124 | pTVC243+chrII-4 (coord. 1024600–1024622); Cm^R^ | | This work |
| pBJH125 | pTVC243+chrII-9 (coord. 1027995–1028019); Cm^R^ | | This work |
| pBJH127 | pTVC243+12-mer (ChrII coord. 572–595); Cm^R^ | | This work |
| pBJH146 | pTVC243+chrII-1 (coord. 956828–956851); Cm^R^ | | This work |
| pBJH147 | pTVC243+chrI-1 (coord. 818082–818105); Cm^R^ | | This work |
| pBJH151 | pMLB1109+VCA1074 promoter (coord. 1027886–1028243); Ap^R^ | | This work |
| pBJH152 | pMLB1109+VCA1075 promoter (coord. 1028243–1027886); Ap^R^ | | This work |
| pBJH154 | pTVC243+chrI-3 (coord. 817800–818099); Cm^R^ | | This work |
| pBJH158 | pTVC243+chrI-2 (coord. 817200–818899); Cm^R^ | | This work |
| pBJH170 | pTVC243+chrI-4 (coord. 817947–818099); Cm^R^ | | This work |
| pBJH174 | pTVC243+chrI-5 (coord. 818000–818099); Cm^R^ | | This work |
| pBJH175 | pTVC243+chrI-10 (coord. 818000–818054); Cm^R^ | | This work |
| pBJH183 | pTVC243+chrI-7 (coord. 818000–818086); Cm^R^ | | This work |
| pBJH184 | pTVC243+chrI-6 (coord. 818010–818099); Cm^R^ | | This work |
| pBJH185 | pTVC243+chrI-8 (coord. 818000–818079); Cm^R^ | | This work |
| pBJH186 | pTVC243+chrI-9 (coord. 818000–818069); Cm^R^ | | This work |
| pBJH188 | pACYC177Δ*bla*::chrI-4 (coord. 817947–818099); Km^R^ | | This work |
| pBJH193 | pMLB1109+VC0765 promoter (coord. 817947–818255); Ap^R^ | | This work |
| pBJH195 | pGB2+chrI-4 (coord. 817947–818099); Sp^R^ | | This work |
| pBJH197 | pTVC31Δ*oriII*::ChrII coord. 818255–817947; Ap^R^ | | This work |
| pBJH223 | pMLB1109+chrI-9 (coord. 818000–818069); Ap^R^ | | This work |
| pBJH227 | pTVC243+chrI-9m (mutated in the footprinted region); Cm^R^ | | This work |
| pBJH228 | pMLB1109+chrI-9m1 (mutated -35 region); Ap^R^ | | This work |
| pBJH229 | pMLB1109+chrI-9m2 (mutated -10 region); Ap^R^ | | This work |
| pBJH230 | pMLB1109+chrI-9m1+m2 (mutated -35 and -10 regions); Ap^R^ | | This work |
| pBJH235 | pMLB1109+chrI-4 (coord. 817947–818099); Ap^R^ | | This work |
| pBJH238 | pTVC243+chrI-4m1 (mutated -35 region); Cm^R^ | | This work |
| pBJH239 | pTVC243+chrI-4m2 (mutated -10 region); Cm^R^ | | This work |
| pBJH240 | pTVC243+ chrI-4m1+m2 (mutated -35 and -10 regions); Cm^R^ | | This work |
| pBJH242 | pEM7-Zeo+upstream flank of chrI-4 (coord. 816950–817954); Ap^R^, Zeo^R^ | | This work |
| pBJH243 | pACYC177Δ*bla*::chrI-6 (coord. 818010–818099); Km^R^ | | This work |
| pBJH245 | pBJH242+downstream flank of chrI-4 (coord. 818104–819099); Ap^R^, Zeo^R^ | | This work |
| pBJH247 | pTVC243+chrI-9m1 (mutated -35 region); Ap^R^ | | This work |
| pBJH248 | pTVC243+chrI-9m2 (mutated -10 region); Ap^R^ | | This work |
| pBJH249 | pTVC243+chrI-9m1+m2 (mutated -35 and -10 regions); Ap^R^ | | This work |
| pBJH251 | pEM7-ZeoΔ*bla*::flanks of pTVC243; Zeo^R^ | | This work |
| pBJH252 | pBJH251+39-mer (coord. 449–487); Zeo^R^ | | This work |
| pBJH253 | pBJH251+chrI-4 (coord. 817947–818099); Zeo^R^ | | This work |
| pBJH260 | pTVC11with a mutation in *rctB* causing *R269S* change; Sp^R^ | | This work |
| pBLO1218 | =pBR-flp-hapR* where *flp* is under λ*c*I857(ts) control; Ap^R^ | | This work |
| pEM7-Zeo | Cloning vector and source of Zeocin cassette; Ap^R^, Zeo^R^ | | Invitrogen |
| pGB2 | pSC101 derivative; Sp^R^ | | [[7](#_ENREF_7)] |
| pJAD9 | λ*cI*_N (1-131)_ | | [[8](#_ENREF_8)] |
| pJJ02 | pTVC11with a C-terminally truncated *rctB* (coord. 1118–2621); Sp^R^ | | [[9](#_ENREF_9)] |
| pJJ112 | pJAD9+*rctB* (coord. 1118–3115); Ap^R^, Km^R^ | | J. Jha |
| pKT25 | Bacterial two-hybrid bait vector; p15A*ori*; Km^R^ | | Euromedex |
| pMLB1109 | Source of promoter-less *lacZ* gene; Ap^R^ | | M. Berman |
| pRN010 | *lacI*^q^P*trc*mCherry-pMT*parB*-*gfp*-P1*parB*; pBR322*ori*; Ap^R^ | | [[10](#_ENREF_10)] |
| pTVC11 | *rctB* (coord. 1118–3115) under P_BAD_; pSC101*ori*; Sp^R^ | | [[11](#_ENREF_11)] |
| pTVC25 | *oriII* (coord. 441–1133); R6K*oriγ*; Ap^R^ | | [[12](#_ENREF_12)] |
| pTVC31 | *oriII* (coord. 775–1133); R6K*oriγ*; Ap^R^ | | [[12](#_ENREF_12)] |
| pTVC35 | pTVC31ΔR6K*oriγ*; Ap^R^ | | [[12](#_ENREF_12),[13](#_ENREF_13)] |
| pTVC126 | pMLB1109+*rctA* promoter (coord. 377–249); Ap^R^ | | [[14](#_ENREF_14)] |
| pTVC210 | *oriII* (coord. 109–1133); R6K*oriγ*; Ap^R^ | | [[13](#_ENREF_13)] |
| pTVC222 | pTVC243+39-mer (coord. 449–487); Cm^R^ | | [[14](#_ENREF_14)] |
| pTVC243 | MCS in a transcription-free island; pBR322*ori*; Cm^R^ | | [[13](#_ENREF_13)] |
| pTVC350 | pTVC243+chrII-10 (coord. 1027996–1028033); Cm^R^ | | [[13](#_ENREF_13)] |
| pTVC500 | pMLB1109+*rctB* promoter (coord. 1049–1133); Ap^R^ | | [[9](#_ENREF_9)] |
| pTVC524 | *oriII* (coord. 775–1094); R6K*oriγ*; Ap^R^ | | [[12](#_ENREF_12)] |
| pUT18C | Bacterial two-hybrid prey vector; Ap^R^ | | Euromedex |

**References**

1. Sozhamannan S, Chattoraj DK (1993) Heat shock proteins DnaJ, DnaK, and GrpE stimulate P1 plasmid replication by promoting initiator binding to the origin. J Bacteriol 175: 3546-3555.

2. Fekete RA, Chattoraj DK (2005) A *cis*-acting sequence involved in chromosome segregation in *Escherichia coli*. Mol Microbiol 55: 175-183.

3. Hu JC, O'Shea EK, Kim PS, Sauer RT (1990) Sequence requirements for coiled-coils: analysis with lambda repressor-GCN4 leucine zipper fusions. Science 250: 1400-1403.

4. Val ME, Skovgaard O, Ducos-Galand M, Bland MJ, Mazel D (2012) Genome Engineering in *Vibrio cholerae*: A Feasible Approach to Address Biological Issues. PLoS Genet 8: e1002472.

5. Yamaichi Y, Fogel MA, Waldor MK (2007) par genes and the pathology of chromosome loss in *Vibrio cholerae*. Proc Natl Acad Sci U S A 104: 630-635.

6. Venkova-Canova T, Baek JH, Fitzgerald PC, Blokesch M, Chattoraj DK (2013) Evidence for Two Different Regulatory Mechanisms Linking Replication and Segregation of *Vibrio cholerae* Chromosome II. PLoS Genet 9: e1003579.

7. Churchward G, Belin D, Nagamine Y (1984) A pSC101-derived plasmid which shows no sequence homology to other commonly used cloning vectors. Gene 31: 165-171.

8. Dibbens JA, Muraiso KM, Chattoraj DK (1997) Chaperone-mediated reduction of RepA dimerization is associated with RepA conformational change. Mol Microbiol 25: 185-195.

9. Jha JK, Demarre G, Venkova-Canova T, Chattoraj DK (2012) Replication regulation of *Vibrio cholerae* chromosome II involves initiator binding to the origin both as monomer and as dimer. Nucleic Acids Res 40: 6026-6038.

10. Kadoya R, Chattoraj DK (2012) Insensitivity of Chromosome I and the Cell Cycle to Blockage of Replication and Segregation of *Vibrio cholerae* Chromosome II. MBio 3: 00067-00012.

11. Pal D, Venkova-Canova T, Srivastava P, Chattoraj DK (2005) Multipartite regulation of rctB, the replication initiator gene of *Vibrio cholerae* chromosome II. J Bacteriol 187: 7167-7175.

12. Venkova-Canova T, Srivastava P, Chattoraj DK (2006) Transcriptional inactivation of a regulatory site for replication of *Vibrio cholerae* chromosome II. Proc Natl Acad Sci U S A 103: 12051-12056.

13. Venkova-Canova T, Chattoraj DK (2011) Transition from a plasmid to a chromosomal mode of replication entails additional regulators. Proc Natl Acad Sci U S A 108: 6199-6204.

14. Venkova-Canova T, Saha A, Chattoraj DK (2012) A 29-mer site regulates transcription of the initiator gene as well as function of the replication origin of *Vibrio cholerae* chromosome II. Plasmid 67: 102-110.
