# Supplementary material for: Chromosome I Controls Chromosome II Replication in Vibrio cholerae
Source: PLoS Genet. 2014 Feb 27;10(2):e1004184. doi: 10.1371/journal.pgen.1004184 (PMC3937223; doi:10.1371/journal.pgen.1004184)
Supplement: Text S1 — Supporting Materials and Methods. (DOCX) [file pgen.1004184.s015.docx]

**Supporting Materials and methods**

**Origin activity analysis**

Fragments with chrII coordinates 1024390 - 1025604 and chrI coordinates 817947 - 818255 were cloned individually into an R6K*oriγ* plasmid, which requires the cognate initiator (π) protein for replication. The resulting plasmids, pBJH118 and pBJH197, respectively, and a plasmid containing *oriII* (pTVC31; positive control) were used to transform an *E. coli* strain (BR8706) carrying p*rctB* (pTVC11) that supplied RctB but no π protein. Appearance of colonies indicated origin activity from the cloned fragments.

**Bacterial two-hybrid assay**

The two-hybrid assay was performed using the bacterial adenylate cyclase two-hybrid (BACTH) system (EUK001, Euromedex) as described [[1](#_ENREF_1)]. Pairs of bait (pKT25 or pBJH93) and prey (pUT18C or pBJH95) plasmids were used to transform *E. coli* BTH101 (=CVC1837) cells. These cells were further transformed with either pGB2 or pBJH195. The transformants were grown overnight at 30°C in L broth with appropriate antibiotics in the presence of 50 or 500 μM IPTG. *β*-galactosidase activity was measured to determine the degree of functional complementation between the fusion proteins.

**λ*P*_R_ repression assay**

The repression assay was performed using the bacteriophage λ repressor as described [[2](#_ENREF_2)]. The *rctB* gene was cloned into pJAD9 in frame with λ*cI*_N_. The resulting plasmid (pJJ112) and the empty vector (pJAD9) were used to transform BR6610 cells. These cells were further transformed with either pGB2 (vector) or the same vector carrying chrI-4 (pchrI-4=pBJH195). The transformants were grown to exponential phase at 37°C in L broth containing appropriate antibiotics and 0, 5 or 50 μM IPTG. *β*-galactosidase activity was measured to determine the degree of repression.

**ATP hydrolysis assay**

The ATP hydrolysis activity of RctB was measured colorimetrically using an ATPase assay kit (Innova Biosciences). After 30 min incubation at room temperature, the reaction mixture containing 100 nM supercoiled DNA (pTVC243, pTVC350 or pBJH170) and RctB (0, 0.5 or 5 μM) was used to measure the absorbance at 630 nm using Synergy HT Microplate Reader (BioTek).

**Quantitative RT-PCR assay**

qRT-PCR was performed using iTaq Universal SYBR Green One-Step Kit (Bio-Rad). Total RNA from *E. coli* Δ*dnaKJ* cells containing p*oriII* (pTVC31), p*rctB* (pTVC11) and either the empty vector (pTVC243) or the same vector carrying chrI-4 (pBJH170) was isolated using RNeasy Mini kit (Qiagen). The level of *rctB* expression was determined with (0.002%) and without arabinose induction in *E. coli*, and in *V. cholerae* WT and *ΔchrI-4* cells.

**References**

1. Venkova-Canova T, Baek JH, Fitzgerald PC, Blokesch M, Chattoraj DK (2013) Evidence for Two Different Regulatory Mechanisms Linking Replication and Segregation of *Vibrio cholerae* Chromosome II. PLoS Genet 9: e1003579.

2. Hu JC, O'Shea EK, Kim PS, Sauer RT (1990) Sequence requirements for coiled-coils: analysis with lambda repressor-GCN4 leucine zipper fusions. Science 250: 1400-1403.
